# Recent Advances in TiO_2_-Based Heterojunctions for Photocatalytic CO_2_ Reduction With Water Oxidation: A Review

**DOI:** 10.3389/fchem.2021.637501

**Published:** 2021-04-15

**Authors:** Kai Li, Chao Teng, Shuang Wang, Qianhao Min

**Affiliations:** ^1^ Institute of Marine Biomedicine, Shenzhen Polytechnic, Shenzhen, China; ^2^ State Key Laboratory of Analytical Chemistry for Life Science, School of Chemistry and Chemical Engineering, Nanjing University, Nanjing, China; ^3^ College of Engineering and Applied Sciences, Nanjing University, Nanjing, China

**Keywords:** TIO_2_-based photocatalysts, heterostructures, CO_2_ photoreduction, water oxidation, high efficiency

## Abstract

Photocatalytic conversion of CO_2_ into solar fuels has gained increasing attention due to its great potential for alleviating the energy and environmental crisis at the same time. The low-cost TiO_2_ with suitable band structure and high resistibility to light corrosion has proven to be very promising for photoreduction of CO_2_ using water as the source of electrons and protons. However, the narrow spectral response range (ultraviolet region only) as well as the rapid recombination of photo-induced electron-hole pairs within pristine TiO_2_ results in the low utilization of solar energy and limited photocatalytic efficiency. Besides, its low selectivity toward photoreduction products of CO_2_ should also be improved. Combination of TiO_2_ with other photoelectric active materials, such as metal oxide/sulfide semiconductors, metal nanoparticles and carbon-based nanostructures, for the construction of well-defined heterostructures can enhance the quantum efficiency significantly by promoting visible light adsorption, facilitating charge transfer and suppressing the recombination of charge carriers, resulting in the enhanced photocatalytic performance of the composite photocatalytic system. In addition, the adsorption and activation of CO_2_ on these heterojunctions are also promoted, therefore enhancing the turnover frequency (TOF) of CO_2_ molecules, so as to the improved selectivity of photoreduction products. This review focus on the recent advances of photocatalytic CO_2_ reduction via TiO_2_-based heterojunctions with water oxidation. The rational design, fabrication, photocatalytic performance and CO_2_ photoreduction mechanisms of typical TiO_2_-based heterojunctions, including semiconductor-semiconductor (S-S), semiconductor-metal (S-M), semiconductor-carbon group (S-C) and multicomponent heterojunction are reviewed and discussed. Moreover, the TiO_2_-based phase heterojunction and facet heterojunction are also summarized and analyzed. In the end, the current challenges and future prospects of the TiO_2_-based heterostructures for photoreduction of CO_2_ with high efficiency, even for practical application are discussed.

## Introduction

Energy and environmental crizes are two major bottlenecks restricting the sustainable development of human society. For a long time, the excessive consumption of fossil fuels has caused severe energy shortages, and the CO_2_ released during the combustion process has become the main factor leading to global warming (Stott et al., 2000; Meinshausen et al., 2009; Solomon et al., 2009; Höök and Tang, 2013). It is urgent to develop and utilize renewable clean energy while reducing the concentration of CO_2_ in the atmosphere (Brockway et al., 2019; Shindell and Smith, 2019). Notably, as a simple form of carbon storage, the rich carbon resources contained in CO_2_ have huge development potential. Using CO_2_ as a carbon feedstock to prepare carbon-based fuels can help alleviate the energy crisis and global warming at the same time, and has become a current research hotspot in the fields of both energy and environment (Olah et al., 2011; Kondratenko et al., 2013; Aresta et al., 2014; Ganesh, 2014; Li et al., 2019). However, the liner molecule with high thermodynamic stability and kinetic inertness makes it a great challenge for the activation and conversion of CO_2_ (Ola and Maroto-Valer, 2015; Wei L. et al., 2018; Li et al., 2019; Nguyen et al., 2020). A lot of energy needs to be injected to break the C=O bond (dissociation energy about 750 kJ mol^−1^) in CO_2_ (Kim et al., 2017). Moreover, the extremely low water solubility of CO_2_ (about 30 mM under 25°C at 1 atm) results in the low conversion efficiency of CO_2_ in the aqueous reaction system (Xie et al., 2014). Therefore, a highly efficient reaction mode is also in great demand.

**FIGURE 1:**
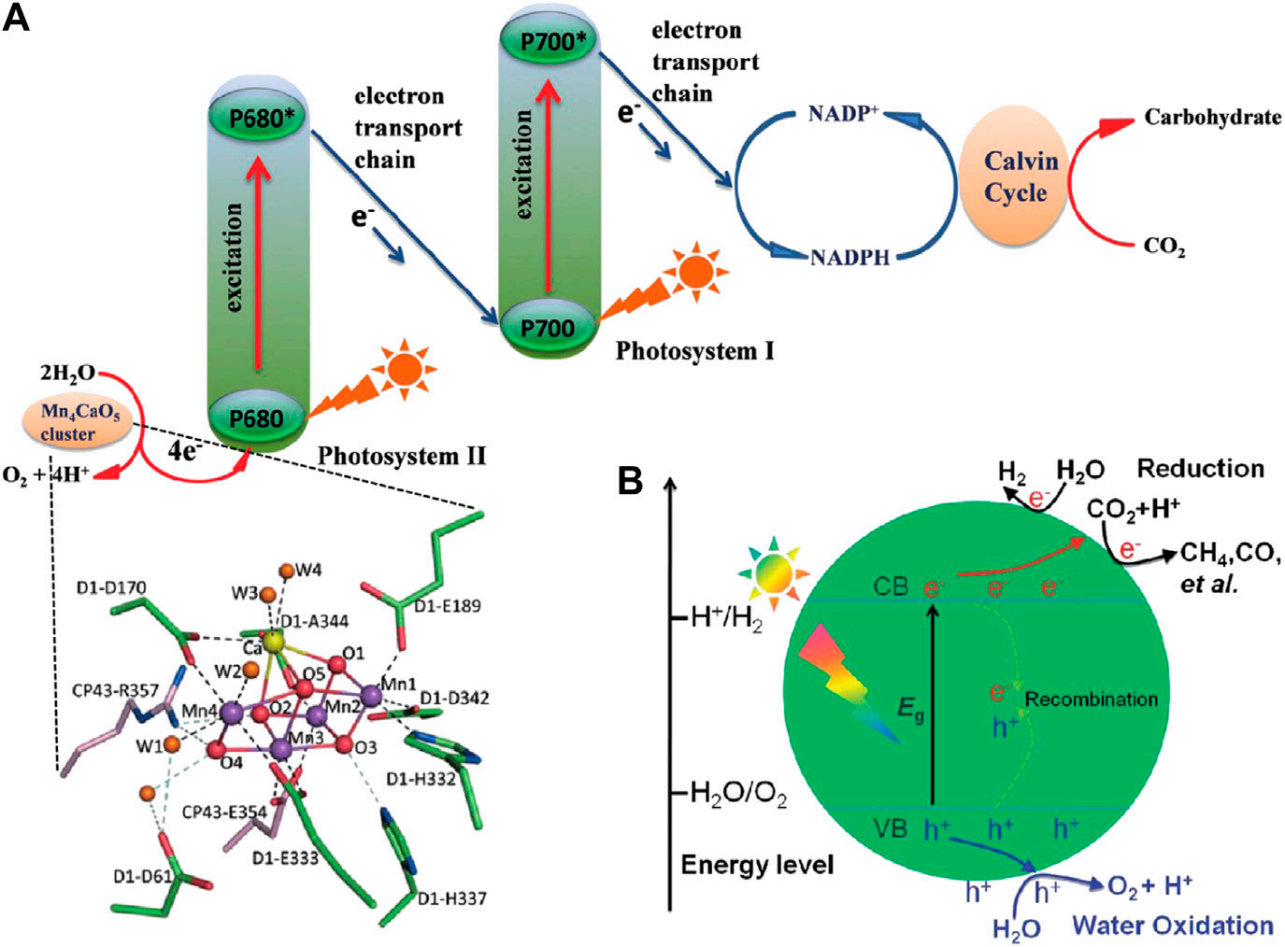
Schematic diagrams of **(A)** natural photosynthesis and **(B)** semiconductor photocatalytic reduction of CO_2_. Reproduced from Liu et al. (2016) with permission from Wiley-VCH and Wei et al. (2018) with permission from the Royal Society of Chemistry.

Fortunately, the natural photosynthesis motivated by solar energy to covert CO_2_ into carbonhydrates as well as the release of O_2_ by water oxidation provides a very promising solution to reduce the CO_2_ level in atmosphere, which inspires the development of artificial photosynthesis systems (Barber, 2009; Dogutan and Nocera, 2019; Zhang and Sun, 2019). As shown in Figure 1A, the water oxidation process takes place in the photosystem II (PSII) of green plants to provide electrons and protons for the CO_2_ fixation and conversion in the photosystem I (PSI). Since a series of pioneering works devoted by Fujishima and Honda on semiconductor photocatalysis in the 1970s (Fujishima and Honda, 1972; Inoue et al., 1979), substantial efforts have been made for the combination of the two individual processes within a single artificial architecture to mimic the natural photosynthesis (shown in Figure 1B) during the past decades (Ma et al., 2014; Tu et al., 2014; White et al., 2015; Liu X. et al., 2016; Li et al., 2019; Xu and Carter, 2019).

In a typical semiconductor photocatalytic process, the semiconductor photocatalyst is excited by the incident photons with energy greater than or equal to its bandgap energy (*E*_*g*_), where electrons (*e* ^*-*^) are excited to the conduction band (CB) to participate in the reduction reactions, remaining holes (*h* ^*+*^) in the valence band (VB) for the oxidation reactions. Considering that CO_2_ molecule is very stable and the photocatalytic CO_2_ reduction is a series of uphill reactions (ΔG^0^>0, see in Table 1) (Wei L. et al., 2018), a large energy input is necessary to covert CO_2_ into solar fuels, corresponding to the photons in the ultraviolet or shortwave visible regions. In addition, the band structure of ideal semiconductors needs to meet the redox potentials of CO_2_ reduction and water oxidation reactions at the same time, as listed in Table 2 (Habisreutinger et al., 2013; White et al., 2015). Generally, the CB edge position (*E*_CB_) should be more negative than the potential for reducing CO_2_, whereas the VB edge position (*E*_VB_) should be more positive than the potential for oxidizing H_2_O to O_2_. So far, the photocatalytic activity of numerous photocatalysts, etc. TiO_2_ (Habisreutinger et al., 2013; Ma et al., 2014; Ola and Maroto-Valer, 2015), ZnO (Lee K. M. et al., 2016; Nie et al., 2018; Kegel et al., 2018), WO_3_ (Chen et al., 2012; Shi et al., 2019; Wang H. et al., 2019), SnO_2_ (He et al., 2015; You et al., 2020), Cu_2_O (An et al., 2014; Shi et al., 2019), CdS (Kuai et al., 2015; Kuehnel et al., 2017; Wei Z. H. et al., 2018; Bie et al., 2019; Low et al., 2019), Bi_2_WO_6_ (Cao et al., 2018; Liu et al., 2021), BiVO_4_ (Mao et al., 2012; Wei Z. H. et al., 2018), BiOBr (Ye et al., 2016; Wu et al., 2018), g-C_3_N_4_ (He et al., 2015; Nie et al., 2018; Thi Thanh Truc et al., 2019; Wang et al., 2020a) and graphene (An et al., 2014; Xiong et al., 2016; Shehzad et al., 2018a; Zhao H. et al., 2018; Bie et al., 2019), have been investigated intensively, in which few of them can realize the synergism of photocatalytic CO_2_ reduction and water oxidation. In particular, the low-cost TiO_2_ with suitable band structure and high resistibility to light corrosion is a very promising candidate, which has become the benchmark in this field (Habisreutinger et al., 2013; Ma et al., 2014; Ola and Maroto-Valer, 2015). However, the wide band gap of TiO_2_ (3.2 eV for anatase) responses to UV light only, which accounts for only 3–5% of the incoming solar spectrum, thus restricting the conversion efficiency of solar energy. Besides, the fast recombination of photo-induced *e* ^*-*^/*h* ^*+*^ pairs within TiO_2_ results in the low charge separation efficiency, therefore reducing its photocatalytic performance further. Moreover, the low selectivity toward photoreduction products of CO_2_ based on aqueous TiO_2_ suspension photocatalytic system should also be improved.

**TABLE 1:** The possible reactions during the photocatalytic CO_2_ reduction process.

	Reactions	Δ*G* ^0^ (kJ∙mol^−1^)
1	$H_{2}O\;\left(l\right)\to H_{2}\;\left(g\right)+1/2O_{2\;}\left(g\right)$	237
2	$CO_{2\;}\left(g\right)\to CO\;\left(g\right)+1/2O_{2\;}\left(g\right)$	257
3	$CO_{2\;}\left(g\right)+H_{2}O\;\left(l\right)\to HCOOH\;\left(l\right)+1/2O_{2\;}\left(g\right)$	286
4	$CO_{2\;}\left(g\right)+H_{2}O\;\left(l\right)\to HCHO\;\left(l\right)+O_{2\;}\left(g\right)$	522
5	$CO_{2\;}\left(g\right)+2H_{2}O\;\left(l\right)\to CH_{3}OH\;\left(l\right)+3/2O_{2}\left(g\right)$	703
6	$CO_{2\;}\left(g\right)+2H_{2}O\;\left(l\right)\to CH_{4}\;\left(g\right)+2O_{2\;}\left(g\right)$	818

**TABLE 2:** Electrochemical potentials of H_2_O oxidation and CO_2_ reduction into various products.

	Reactions	*E* ^0^ (V) *vs*. NHE at pH 7
1	$2H_{2}O+4h^{+}\to O_{2}+4H^{+}$	1.23
2	$CO_{2}+e^{-}\to CO_{2}^{-}$	−1.9
3	$CO_{2}+2H^{+}+2e^{-}\to CO+H_{2}O$	−0.53
4	$CO_{2}+2H^{+}+2e^{-}\to HCOOH$	−0.61
5	$CO_{2}+4H^{+}+4e^{-}\to HCHO+H_{2}O$	−0.48
6	$CO_{2}+6H^{+}+6e^{-}\to CH_{3}OH+H_{2}O$	−0.38
7	$CO_{2}+8H^{+}+8e^{-}\to CH_{4}+2H_{2}O$	−0.24
8	$2H^{+}+2e^{-}\to H_{2}$	−0.41

In the past few decades, various of strategies have been developed to enhance the photocatalytic performance of TiO_2_. Among them, the nanostructured TiO_2_ with single crystalline phase exhibited the decreased recombination rate of charge carriers, comparing to the polycrystalline samples that possess large amount of grain boundaries and defects acting as recombination centers (Tu et al., 2014; Xu et al., 2015). Moreover, crystal facet engineering has been adopted to tune the surface energy and active sites of TiO_2_, contributing to the adsorption and activation of CO_2_ (Liu L. et al., 2016; Xiong et al., 2018; Tu et al., 2020). Obviously, the preference adsorption of CO_2_ molecules at the surface oxygen vacancy sites of TiO_2_ can reduce the reactive barrier of CO_2_ photoreduction reactions, in which one oxygen atom of CO_2_ is trapped by a bridging oxygen vacancy defect to induce affinity interactions (Lee et al., 2011; Liu L. et al., 2016; Tan et al., 2017). Moreover, the localized electrons of oxygen vacancies can form adventitious energy levels, extending the photoresponsive range of semiconductor photocatalyst. Besides, surface oxygen vacancies with typical defect states can trap electrons or holes to inhibit their recombination (Wang et al., 2018). To sum up, the significance of surface oxygen vacancies on defected TiO_2_ has been ascertained in the enhancement of CO_2_ adsorption, activation, dissolution, and stabilization of reaction intermediates (Nguyen et al., 2020). In addition, metal/nonmetal ion doping is used to introduce additional energy level between the band gap of TiO_2_, resulting in the reduced band width and enhanced visible light adsorption (Tu et al., 2014; Ola and Maroto-Valer, 2015; Shehzad et al., 2018b; Patil et al., 2019). Dye sensitized TiO_2_ displays enhanced photoreduction efficiency of CO_2_ due to the injection of photosensitized electrons from the energy level of dye molecule to the CB of TiO_2_ with more negative potential, while the superior visible light responsibility of dye molecules can also improve the utilization of incident light (Ma et al., 2014; Ola and Maroto-Valer, 2015; Lee J. S. et al., 2016; Woo et al., 2019). Although these strategies have proven to be effective, the charge separation efficiency, light energy utilization and product selectivity still need to be further improved to fulfill the demand of more efficient photoreduction of CO_2_, even for the practical application in the future.

As is known, construction of heterojunction between TiO_2_ and cocatalyst with matching electronic band structures can significantly promote the separation of photogenerated *e* ^*-*^ and *h* ^*+*^, enlarge the spectra response range, while the physicochemical properties of some special cocatalyst can promote the photocatalytic CO_2_ reduction or water oxidation to a certain extent, thereby resulting in high photoreduction efficiency of CO_2_ over the heterostructured phocatalytic system with enhanced reduction products selectivity (Ma et al., 2014; Wang et al., 2014; Wei L. et al., 2018; Nguyen et al., 2020).

In addition to photocatalysts, photoreactors as well as reaction modes also play vital roles in affecting the photoreduction efficiency of CO_2_. Generally, the two key parameters which determine the types of photoreactors utilized in CO_2_ photoreduction are the phases involved (i.e., gas-solid, liquid-solid) and the mode of operation (i.e., batch, semi-batch or continuous). In the solid-liquid cases, photocatalysts are usually dispersed in alkaline mediums (aqueous solution) which can realize higher CO_2_ solubility, resulting in the formation of CO_3_^2−^ and HCO_3_^−^. However, these species are difficult to be reduced in comparison with CO_2_ (Corma and Garcia, 2013). In order to overcome the above drawbacks, the solid-vapor mode has been widely applied where the generation rate of the products for CO_2_ photoreduction can be improved significantly (Xie et al., 2014; Xie et al., 2016; Xiong et al., 2017b). In addition, the exposure of photocatalysts in a CO_2_ atmosphere can reduce the generation of H_2_, thus enhancing the selectivity for CO_2_ reduction. Obviously, the solid-vapor mode is more suitable for CO_2_ photoreduction in the presence of H_2_O.

**FIGURE 2:**
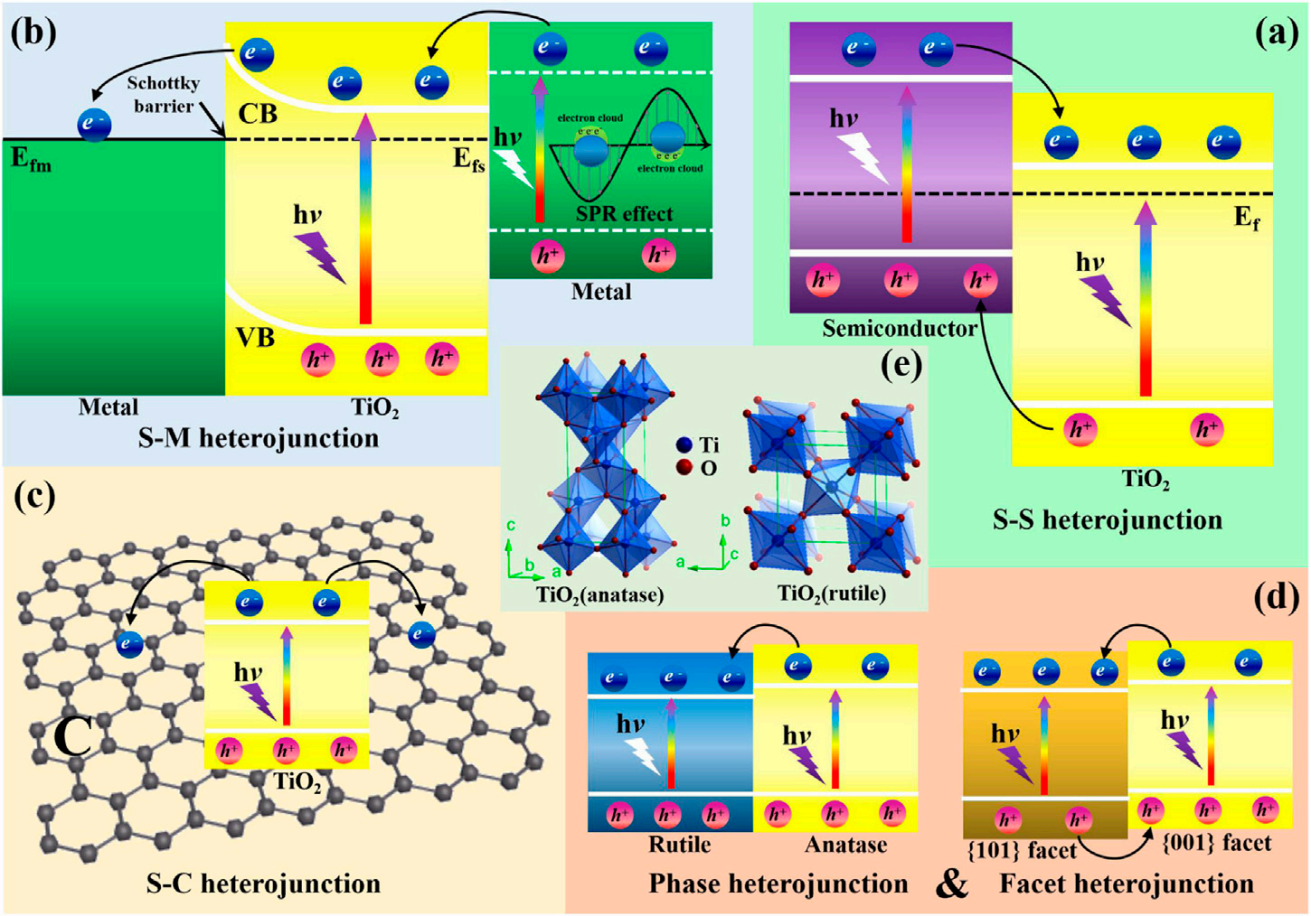
Separation and transfer of photogenerated charge carriers in the TiO_2_-based **(A)** S-S heterojunction, **(B)** S-M heterojunction, **(C)** S-C heterojunction and **(D)** phase and facet heterojunction; **(E)** Crystalline structures of TiO_2_ (anatase) and TiO_2_ (rutile).

In this review, we will mainly focus on the recent advances of photocatatytic CO_2_ reduction processes with water oxidation using TiO_2_-based heterojunction as photocatalysts. Different categories of heterojunctions, including S-S heterojunction (Figure 2A), S-M heterojunction (Figure 2B), S-C heterojunction (Figure 2C), multicomponent heterojunction, phase heterojunction and facet heterojunction (Figure 2D) are reviewed individually. In addition, the unique functions of cocatalysts among different heterostructured photocatalytic systems (etc. photosensitizer, CO_2_ reduction promoter, water oxidation promoter and surface plasmon resonance (SPR) source) as well as the photoreduction mechanisms of CO_2_ are discussed in detail. In the end, we will look forward to the prospects, opportunities and challenges of photocatalytic CO_2_ reduction, predict the research directions of this field in the future, and put forward our opinions on the construction of efficient multifunctional integrated photocatalytic CO_2_ reduction systems. We believe that this review will provide some useful guidelines for the construction of heterostructured photocatalysts for photoreduction of CO_2_ with high performance in the future.

## Photoreduction of CO_2_ to Solar Fuels on TiO_2_-Based Heterojunctions

From the perspective of semiconductor photocatalysis, the premise of high photocatalytic efficiency is the efficient separation and rapid transfer of photogenerated *e* ^*-*^/*h* ^*+*^ pairs, thereby prolonging their lifetimes and inhibiting their recombination. The strategy for the construction of heterojunction by coupling semiconductor (TiO_2_) with a secondary substance including other semiconductors (Aguirre et al., 2017; Yang et al., 2017; She et al., 2018; Xu et al., 2018a; Jin et al., 2019; Low et al., 2019; Thi Thanh Truc et al., 2019; Wu et al., 2019), metal nanoparticles (Xie et al., 2013; Neaţu et al., 2014; Jiao et al., 2015; Xiong et al., 2015; Lee K. Y. et al., 2016; Yu et al., 2016; Cheng et al., 2017; Tahir et al., 2017; Xiong et al., 2017C; Chong et al., 2018; Low et al., 2018; Tan et al., 2018; Tasbihi et al., 2018a; Wei Y. et al., 2018; Zhao Y. et al., 2018; Khatun et al., 2019; Wang R. et al., 2019; Zeng et al., 2020; Ziarati et al., 2020; Wang et al., 2021) and carbon-based nanostructures (Xia et al., 2007; Tu et al., 2013; Gui et al., 2014; Chowdhury et al., 2015; Xiong et al., 2016; Lin et al., 2017; Tan et al., 2017; Wang et al., 2017; Li et al., 2018; Olowoyo et al., 2018; Shehzad et al., 2018a; Zhang J. et al., 2018; Zubair et al., 2018; Bie et al., 2019; Olowoyo et al., 2019; Wang R. et al., 2019; Rodríguez et al., 2020) has been generally applied. Since different phases (etc. anatase, brookite, or rutile) and exposed facets (etc. (001) or (101)) of TiO_2_ exhibit various of band structure and reactivity, TiO_2_-based phase heterojunction (Reñones et al., 2016; Hwang et al., 2019; Jin et al., 2019; Chen et al., 2020; Lee J. S. et al., 2016; Xiong et al., 2020) or facet heterojunction (Yu et al., 2014; Cao et al., 2016; Liu L. et al., 2016; Xiong et al., 2016; Xiong et al., 2017a; Di Liberto et al., 2019) are also fabricated for photocatalytic CO_2_ reduction, which exhibits enhanced photocatalytic efficiency in comparison with pristine TiO_2_. On the one hand, the heterostructure facilitates the separation of photoinduced charge carriers which then transfer across the heterointerface to restrain recombination. On the other hand, the additional active sites introduced by the cocatalysts favor for the adsorption and activation of CO_2_, thus enhancing the photoreduction efficiency of CO_2_. Besides, the promoted quantum efficiency and product selectivity can also be expected by the constructed heterojunctions, since light-excitation attributes, band structure, and separation efficiency of photogenerated charge carriers of heterojunctions play vital roles in the selectivity of CO_2_ photoreduction products. Moreover, various cocatalysts with different reactive active sites can also affect the product selectivity greatly, where the adsorption/activation of CO_2_ as well as the adsorption/desorption of the intermediates are tuned (Fu et al., 2019)**.**

In this section, the rational design, fabrication, photocatalytic performance and photoreduction mechanism of CO_2_ over the TiO_2_-based typical categories of heterojunctions (S-S, S-M, S-C, multicomponent, phase and facet heterojunction) will be reviewed and discussed in detail. In addition, the selectivity toward photoreduction products is another focus. The relative mechanism was concluded and analyzed in the certain case.

### TiO_2_ Based Z-scheme S-S Heterojunction for CO_2_ Photoreduction

**FIGURE 3:**
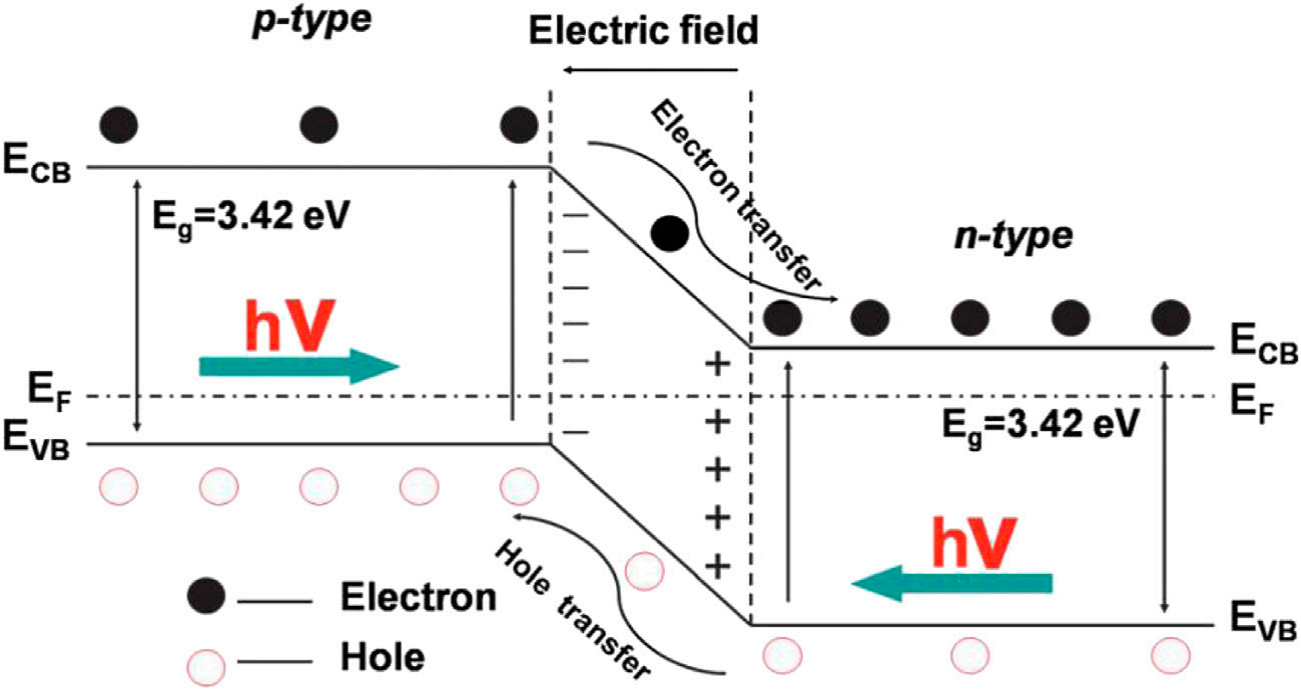
Schematic diagram showing the energy band structure and electron-hole pair separation in the p-n heterojunction. Reproduced from Wang et al. (2014) with permission from the Royal Society of Chemistry.

**FIGURE 4:**
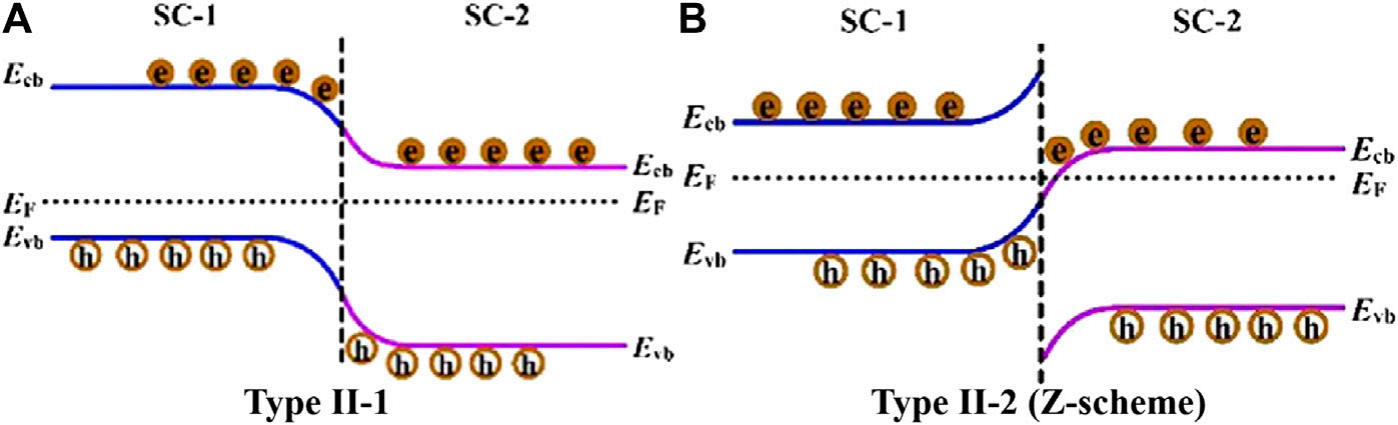
Photogenerated charge carrier transfer process for two types of non p-n heterojunctions: **(A)** type II-1, and **(B)** type II-2 (direct Z-scheme). Reproduced from Zhang and Jaroniec (2018) with permission from Elsevier and Copyright Clearance Center.

Coupling n-type TiO_2_ with a p-type semiconductor possessing matching energy band structure to form a p-n heterojunction is one of the most classic S-S heterojunction (Zeng et al., 2014; Aguirre et al., 2017; Lee et al., 2017; Zhang L. et al., 2018; Iqbal et al., 2020; Zhang et al., 2020). As shown in Figure 3, the contacting of the two semiconductors leads to the diffusion of *e* ^*-*^ and *h* ^*+*^, then forms a space-charge region at the interface of the p-n heterojunction (Wang et al., 2014). As a result, a strong built-in electrical field is created which can drive the photoinduced *e* ^*-*^ and *h* ^*+*^ to transfer in the opposite directions, therefore enhancing the separation efficiency of charge carriers. In addition to p-n heterojunction, TiO_2_-based non-p-n heterojunctions are also common (Yang et al., 2017; She et al., 2018; Jin et al., 2019; Low et al., 2019; Thi Thanh Truc et al., 2019; Wu et al., 2019). Typically, two closely integrated semiconductors with staggered band configurations can form a type II-1 heterojunction (shown in Figure 4A) (Zhang and Jaroniec, 2018), in which the band bending facilitates the charge transfer at the heterointerface. Specifically, *e* ^*-*^ and *h* ^*+*^ are separated individually in both semiconductor 1 (SC-1) and semiconductor 2 (SC-2) under the irradiation of incident light. The difference in energy level leads to the transfer of *e* ^*-*^ from the CB of SC-1 with more negative potential to the CB of SC-2. Meanwhile, *h* ^*+*^ can transfer from the VB of SC-2 to the VB of SC-1 with more positive potential. Similar to the p-n heterojunction, the reverse migration of *e* ^*-*^ and *h* ^*+*^ in the type II-1 heterojunction improves the separation efficiency of charge carriers, thus endowing the enhanced photocatalytic performance of the heterostructured system. However, the way of carrier transfer in the above heterojunctions will lead to a decrease in their redox ability, making it difficult to ensure the optimal photocatalytic activity.

Recently, study on the construction of all-solid-state Z-scheme heterojunction has gained great attention of researchers (Kuai et al., 2015; Aguirre et al., 2017; Takayama et al., 2017; Yang et al., 2017; She et al., 2018; Low et al., 2019; Thi Thanh Truc et al., 2019; Wang et al., 2019; Raza et al., 2020; Wang et al., 2020). Comparing to the p-n and type II-1 heterojunctions, the carrier transfer mode in the Z-scheme heterojunction is more favorable for photocatalytic application. In general, the band bending at the interface of direct Z-scheme heterojunction (type II-2) is conducive to the recombination of photoinduced *e* ^*-*^ and *h* ^*+*^ with weaker reduction and oxidation ability, so that *e* ^*-*^ in the more negative CB of SC-1 and *h* ^*+*^ in the more positive VB of SC-2 can be remained (shown in Figure 4B). As a result, both high separation efficiency and optimal redox ability of photoinduced charge carriers can be realized, thus endowing the high photocatalytic performance of the Z-scheme system. In this section, recent advances for the construction of TiO_2_-based all-solid-state indirect and direct Z-scheme heterojunctions as well as their application for photocatalytic CO_2_ reduction with water oxidation will be reviewed and discussed in detail. Photocatalytic CO_2_ reduction performance of the typical TiO_2_-based all-solid-state Z-scheme heterojunctions are listed in Table 3.

**TABLE 3:** Photocatalytic CO_2_ reduction performance on typical TiO_2_-based S-S (Z-scheme), S-M, S-C, multicomponent, phase and facet heterojunctions.

Photocatalyst	Reductant	Light source	Generation rate of main products (μmol∙g_cat_^−1^∙h^−1^)	Quantum efficiency (%)	References
Indirect Z-scheme heterojunction					
CdS/rGO/TiO_2_	H_2_O vapor	300 W	CH_4_: 0.12 (μmol∙h^−1^)	-	Kuai et al. (2015)
		Xe lamp			
CuGaS_2_-RGO-TiO_2_	Na_2_S aqueous solution	300 W	CO: 0.15	-	Takayama et al. (2017)
		Xe lamp (λ > 330 nm)	H_2_: 28.8 (μmol∙h^−1^)		
Al−O Linked porous-g-C_3_N_4_/TiO_2_-nanotube (PCN/TNT)	Na_2_SO_4_ aqueous solution	150 W Xe lamp	CH_3_COOH	-	Wu et al. (2019)
			HCOOH		
			CH_3_OH		
ZnFe_2_O_4_/Ag/TiO_2_ nanorods	H_2_O vapor	200 W Hg lamp	CO: 1025	-	Tahir (2020)
			CH_4_: 132		
			CH_3_OH: 30.8		
			C_2_H_6_: 19.1 (μmol∙h^−1^)		
g-C_3_N_4_/Pt/3DOM-TiO_2_@C	H_2_O vapor	300 W Xe lamp (λ ≥ 420 nm)	CO: 1.47	5.67	Wang et al. (2020a)
			CH_4_: 6.56		
			H_2_: 0.82		
(Au/A-TiO_2_)@g-C_3_N_4_	H_2_O vapor	300 W Xe lamp (λ ≥ 420 nm)	CH_4_: 37.4	1.91	Wang et al. (2020b)
			CO: 21.7		
Direct Z-scheme heterojunction					
Cu_2_O/TiO_2_	H_2_O vapor	1 kW high-pressure Hg (Xe) arc lamp (λ ≥ 305 nm)	CO: 2.11	-	Aguirre et al. (2017)
ZnIn_2_S_4_/TiO_2_	H_2_O vapor	300 W Xe lamp	CH_4_: 1.135	-	Yang et al. (2017)
TiO_2_/CuInS_2_	H_2_O vapor	350 W Xe lamp	CH_4_: 2.5	-	Xu et al. (2018b)
TiO_2_/CdS	H_2_O vapor	300 W Xe lamp	CH_4_: 11.9	-	Low et al. (2019)
			μmol∙h^−1^∙m^−2^		
Zn_3_In_2_S_6_/TiO_2_	H_2_O vapor	300 W Xe lamp	CH_4_: 6.19	-	She et al. (2018)
			CO: 23.35		
Nb-TiO_2_/g-C_3_N_4_	H_2_O vapor	Two 30 W white bulbs	CH_4_: 562	-	Thi Thanh Truc et al. (2019)
			CO: 420		
			HCOOH: 698		
Copper (II)-porphyrin zirconium metal-organic framework (PCN-224(Cu))/TiO_2_	Na_2_SO_4_ aqueous solution	300 W Xe lamp	CO: 37.21	-	Wang L. et al. (2019)
WO_3_-TiO_2_/Cu_2_ZnSnS_4_	H_2_O vapor	400 W Xe lamp (λ > 420 nm)	CH_4_: 1.69	0.52	Raza et al. (2020)
			CO: 15.37		
Au-TiO_2_	H_2_O vapor	AM1.5 G simulated sunlight	CH_4_: 302	-	Zeng et al. (2020)
		50 W white cold LED light (λ > 400 nm)	HCHO: 420	-	
			CO: 323		
Single metal					
3DOM Au/TiO_2_	H_2_O vapor	300 W Xe lamp	CH_4_: 2.89	-	Jiao et al. (2015)
Pt^2+^-Pt^0^/TiO_2_	H_2_O vapor	300 W Xe lamp	H_2_: 394.7	0.36	Xiong et al. (2015)
			CH_4_: 37.78		
			CO: 8.03		
Ag/TiO_2_	H_2_O vapor	300 W Xe lamp	CH_4_: 1.40	0.16 (400 nm); 0.013 (520 nm)	Yu et al. (2016)
Ag/TiO_2_ nanorod arrays	H_2_O vapor	300 W Xe lamp (λ > 420 nm)	CH_4_: 1.13	-	Cheng et al. (2017)
			CO: 12		
Pt/TiO_2_	H_2_O vapor	Four 6 W lamps (λ ≤ 365 nm)	CH_4_	-	Tasbihi et al. (2018a)
Pt/TiO_2_-COK-12			CO		
Ag/TiO_2_ nanotube arrays (TNTAs)	H_2_O vapor	300 W Xe lamp	CH_4_	-	Low et al. (2018)
Pt/TiO_2_-Al_2_O_3_ foam	H_2_O vapor	UV 8 W Hg lamp	H_2_: 22.5	-	Tasbihi et al. (2018b)
			CH_4_: 1.21		
			CO: 0.54		
Au-TiO_2_ Nanotubes (TNTs)	H_2_O vapor	300 W Xe lamp	CH_4_: 14.67%	-	Khatun et al. (2019)
Au/TiO_2_	H_2_O vapor	300 W Xe lamp	CH_4_: 70.34	-	Wang R. et al. (2019)
			CO: 19.75		
Au/TiO_2_	H_2_O vapor	300 W Xe lamp	CH_4_: 0.2	-	Wang et al. (2021)
			CO: 1.2		
Metal alloy					
(Au, Cu)/TiO_2_	H_2_O vapor	AM1.5 G simulated sunlight	H_2_: 286	-	Neaţu et al. (2014)
			CH_4_: 2200 ± 300		
AgPd/TiO_2_	Triethylamine (TEA) aqueous solution	300 W Xe lamp	H_2_: 144.5	-	Tan et al. (2018)
			CH_4_: 79.0		
PtRu/TiO_2_	H_2_O vapor	300 W Xe lamp	H_2_: 16.5	0.98	Wei Y. et al. (2018)
			CH_4_: 38.7		
			CO: 2.6		
Hierarchical urchin-like yolk@shell TiO_2-x_H_x_ (HUY@S-TOH)/AuPd	H_2_O (liquid)	300 W Xe lamp	CH_4_: 47.0	-	Ziarati et al. (2020)
Graphene and its derivatives					
Graphene-TiO_2_	H_2_O vapor	300 W Xe lamp	CH_4_: 8	-	Tu et al. (2013)
			C_2_H_6_: 16.8		
RGO/Pt-TiO_2_ nanotubes (TNTs)	H_2_O vapor	500 W tungsten-halog--en lamp	CH_4_: 10.96 (μmol∙m^−2^)	-	Sim et al. (2015)
TiO_2_/Nitrogen doped rGO (NrGO)	H_2_O vapor	400 W Xe lamp	CO: 50	0.0072	Lin et al. (2017)
GO/oxygen rich TiO_2_ (OTiO_2_)	H_2_O vapor	300 W Xe lamp	CH_4_: 0.43	0.0103	Tan et al. (2017)
rGO/TiO_2_	H_2_O vapor	500 W Hg lamp	CH_4_: 12.75	-	Shehzad et al. (2018a)
			CO: 11.93		
((Pt/TiO_2_)@rGO)	H_2_O vapor	300 W Xe lamp	H_2_: 5.6	1.93	Zhao Y. et al. (2018)
			CH_4_: 41.3		
			CO: 0.4		
Graphene quantum dots (GQDs)/TiO_2_	H_2_O vapor	100 W Xe solar simulator	CH_4_: 1.98 (ppm∙cm^−2^∙h^−1^)	-	Zubair et al. (2018)
rGO/TiO_2_	Triethanolamine (TEOA) aqueous solution	8 W UV-A lamp	CH_3_OH: 2330	-	Olowoyo et al. (2019)
CNT					
MWCNT/TiO_2_	H_2_O vapor	15 W UV lamp	CH_4_: 11.74	-	Xia et al. (2007)
			HCOOH: 18.67		
			C_2_H_5_OH: 29.87		
MWCNT/TiO_2_	H_2_O (liquid)	15 W energy saving light bulb	CH_4_: 0.17	-	Gui et al. (2014)
Ag-MWCNT@TiO_2_	H_2_O vapor	15 W energy saving light bulb	CH_4_: 0.91	-	Gui et al. (2015)
			C_2_H_6_: 0.048		
MWCNT/TiO_2_	TEOA aqueous solution	8 W UV-A lamp	H_2_: 2360.0	-	Olowoyo et al. (2019)
			CH_3_OH: 3246.1		
			HCOOH: 68.5		
CNT/TiO_2_/Cu	H_2_O vapor	300 W Xe lamp	CH_4_: 1.1	-	Rodríguez et al. (2020)
			CO: 8.1		
Other carbon forms					
Carbon@TiO_2_ hollow spheres	H_2_O vapor	300 W Xe lamp	CH_4_: 4.2	-	Wang et al. (2017)
			CH_3_OH: 9.1		
N, S-containing carbon quantum dots (NCQDs)/TiO_2_	H_2_O vapor	300 W Xe lamp	CH_4_: 0.13	-	Li et al. (2018)
			CO: 0.19		
Carbon nanofibers@TiO_2_	H_2_O vapor	350 W Xe lamp	CH_4_: 13.52	-	Zhang J. et al. (2018)
MgO-Pt-TiO_2_	H_2_O vapor	100 W Xe lamp	H_2_: 14	-	Xie et al. (2014)
			CH_4_: 1.2		
			CO: 1.8		
Pt-rGO-TiO_2_	H_2_O vapor	15 W energy saving light bulb	CH_4_: 0.28	-	Tan et al. (2015)
Pd-rGO-TiO_2_			CH_4_: 0.20		
Ag-rGO-TiO_2_			CH_4_: 0.17		
Au-rGO-TiO_2_			CH_4_: 0.13		
Pt-Cu_2_O/TiO_2_	H_2_O vapor	300 W Xe lamp	CH_4_: 1.42	-	Xiong et al. (2017c)
			CO: 0.05		
WSe_2_-Graphene-TiO_2_	Na_2_SO_3_ aqueous solution	300 W Xe lamp	CH_3_OH: 6.33	-	Biswas et al. (2018)
Pt/MgAl layered double oxides (MgAl-LDO)/TiO_2_	H_2_O (liquid)	300 W Xe lamp	CH_4_: 1.42	-	Chong et al. (2018)
			CO: 2.3		
TiO_2_-Graphene few-layered MoS_2_	H_2_O vapor	300 W Xe lamp	CO: 92.33	-	Jung et al. (2018)
Au/Al_2_O_3_/TiO_2_	H_2_O vapor	450 W Xe lamp	CO: 11.8	-	Zhao H. et al. (2018)
TiO_2_-MnO_x_-Pt	H_2_O vapor	350 W Xe lamp	CH_4_: 34.67	-	Meng et al. (2019)
			CH_3_OH: 30.33 (μmol∙m^−2^∙h^−1^)		
Ag-MgO-TiO_2_	H_2_O vapor	300 W Xe lamp	CH_4_: 0.86	0.091	Xu et al. (2018a)
			CH_3_OH: 0.06		
Au@TiO_2_ hollow spheres (THS)@CoO	H_2_O vapor	300 W Xe lamp	CH_4_: 13.3	-	Zhu et al. (2019)
Phase heterojunction					
Anatase-rutile TiO_2_ fibers	H_2_O vapor	Four 6 W	CO: 10.19	0.036	Reñones et al. (2016)
		UV lamps	CH_4_: 1.34		
			H_2_: 19.94		
Anatase-rutile TiO_2_ nanoparticles with oxygen vacancy	H_2_O vapor	300 W	CH_4_: 43.2	-	Xiong et al. (2020)
		Xe lamp			
Disordered Anatase/ordered rutile (A_d_/R_o_) TiO_2_ nanoparticles	H_2_O vapor	Solar simulator 1 Sun	CH_4_: 3.98	0.273	Hwang et al. (2019)
			CO: 3.02		
Pt-loaded anatase-rutile TiO_2_ nanoparticles	H_2_O vapor	200 W Hg–Xe light	CH_4_	-	Lee et al. (2016)
			CO		
N-doped carbon coating paragenetic anatase/rutile heterojunction	TEOA and MeCN	300 W	CO: 24.31	-	Chen et al. (2020)
		Xe lamp			
SrCO_3_-Modified brookite/anatase TiO_2_ heterojunction	H_2_O vapor	300 W	CH_4_: 19.66	-	Jin et al. (2019)
		Xe lamp	CO: 2.64		
Facet heterojunction					
{101}/{001} TiO_2_	H_2_O vapor	300 W	CH_4_: 1.35	-	Yu et al. (2014)
		Xe lamp			
Oxygen-deficient {101}/{001} TiO_2_	H_2_O vapor	100 W Hg lamp/450 W Xe lamp	CO: ∼10.91 (UV-vis)	0.31 (UV-vis)	Liu L. et al. (2016)
			CO: ∼5.36 (visible)	0.134 (visible)	
Pt-loaded {101}/{001} TiO_2_	0.1 M KHCO_3_ solution	250 W Hg lamp	CH_4_: 4.0	-	Cao et al. (2016)
Pt-loaded {101}/{001} TiO_2_	H_2_O vapor	300 W	CH_4_: 4.6	-	Xiong et al. (2017a)
		Xe lamp	H_2_: 9.9		
Graphene supported {101}/{001} TiO_2_	H_2_O vapor	300 W	CO: 70.8	CO: 0.0557 CH_4_: 0.0864	Xiong et al. (2016)
		Xe lamp	CH_4_: 27.4		

**FIGURE 5:**
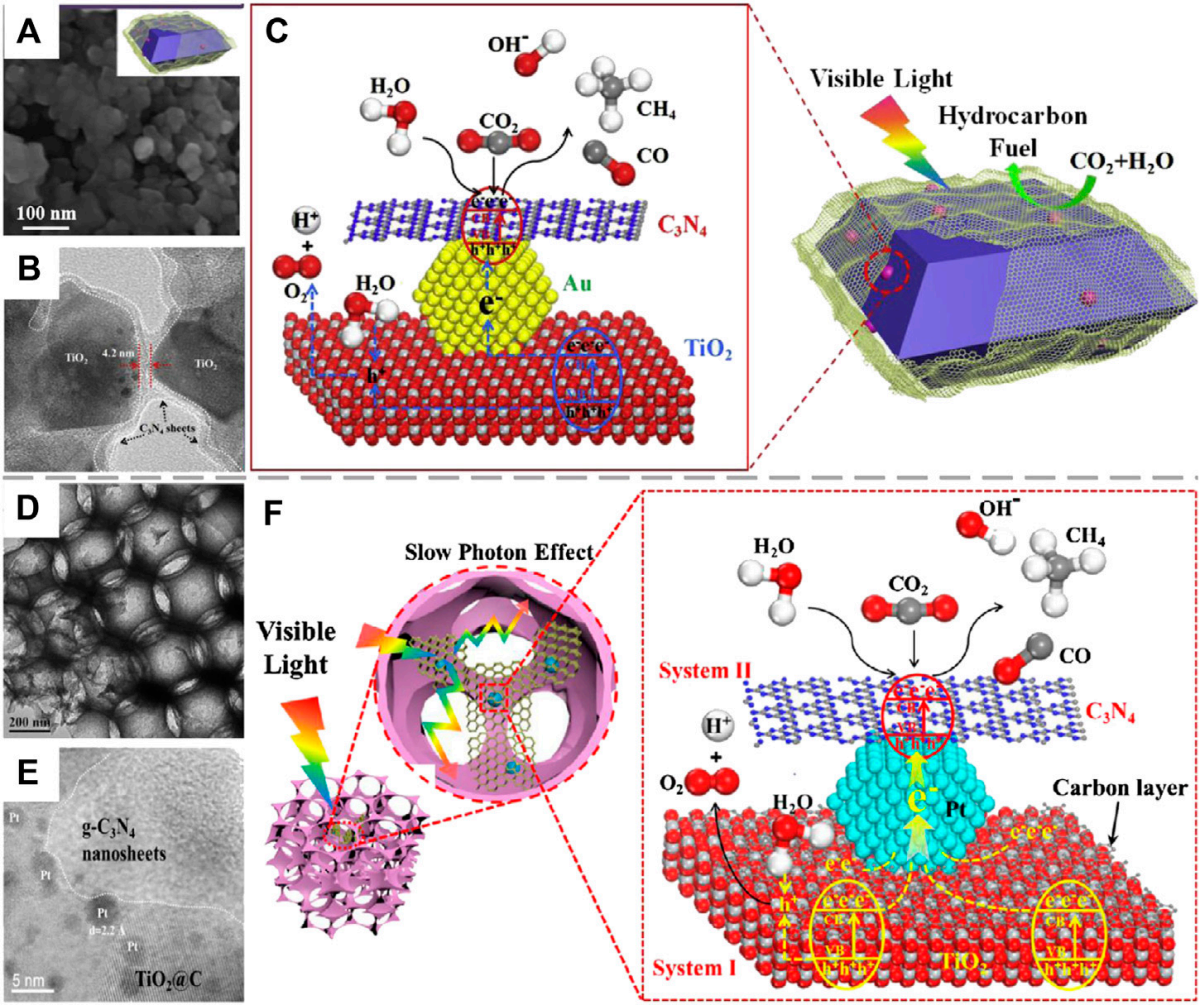
**(A)** SEM and **(B)** HRTEM images of (Au/A-TiO_2_)@g-C_3_N_4_ catalyst; **(C)** Schematic for photorecatalytic CO_2_ reduction with H_2_O to produce CH_4_ and CO over (Au/A-TiO_2_)@g-C_3_N_4_. Reproduced from Wang et al., 2020a with permission from Elsevier and Copyright Clearance Center. **(D)** TEM and **(E)** HRTEM images of 3DOM-CNPTC photocatalyst; **(F)** The mechanism of 3DOM-CNPTC catalyst for photocatalytic CO_2_ reduction with H_2_O to CH_4_. Reproduced from Wang et al. (2020) with permission from Elsevier and Copyright Clearance Center.

Construction of indirect Z-scheme system between TiO_2_ and another semiconductor using noble metals such as Pt (Wang et al., 2020a), Au (Wang et al., 2020b) and Ag (Tahir, 2020) as electron mediators has gained increased attention due its enhanced separation efficiency of photogenerated *e* ^−^/*h* ^+^ pairs with the recombination of inefficient charge carriers, thereby improving the photoreduction efficiency of CO_2_. As reported by Tahir, ZnFe_2_O_4_/Ag/TiO_2_ nanocomposite was fabricated by physical mixing Ag/TiO_2_ nanorods and ZnFe_2_O_4_ nanospheres in methanol solution under continuous stirring (Tahir, 2020). Compared to the point contact between 0D TiO_2_ nanoarticles and 0D ZnFe_2_O_4_ nanospheres, the stronger interaction between 0D ZnFe_2_O_4_ nanospheres and 1D TiO_2_ nanorods is beneficial to the transfer of photogenerated electrons and holes at the interface. At the same time, the migration of these charge carriers along the 1D nanostructure is more efficient, which significantly inhibits their recombination. Moreover, the UV irradiation induced Z-scheme carrier transfer pathway ensures the high redox capability of the remaining carriers with the recombination of inefficient species within Ag nanoparticles, resulting in the superior CO generation rate of 1025 μmol g_cat_^−1^ h^−1^. Compared to ZnFe_2_O_4_, graphic-C_3_N_4_ (g-C_3_N_4_) is more preferred for the construction of TiO_2_-based Z-scheme heterojunction due to its fully visible light utilization, improved CO_2_ adsorption capacity (derived from its surface π bond) and proper band structure for CO_2_ photoreduction with H_2_O oxidation (Wu et al., 2019; Wang et al., 2020a). Moreover, it can also trap photogenerated electrons to enhance charge separation efficiency within the heterojunction. On this basis, g-C_3_N_4_ was coated on the surface of Au/TiO_2_ hybrid to form a Z-scheme photocatalyst (Figure 5A,B) for visible-light-driven (VLD) photocatalytic CO_2_ reduction (Wang et al., 2020a). In particular, the efficient separation of photogenerated *e* ^−^/*h* ^+^ pairs within anatase TiO_2_ is attributed to the formation of {001}/{101} facet heterojunction. Then, photogenerated electrons in the CB of TiO_2_ are directionally transferred through Au and recombine with photogenerated holes in the VB of g-C_3_N_4_, thereby boosting the photoreduction of CO_2_ by photogenerated electrons in the CB of g-C_3_N_4_ (shown in Figure 5C). Notably, the high selectivity toward CO_2_ photoreduction (>99%) was realized with few H_2_ generation, while the selectivity for CH_4_ generation (63.3%) was also enhanced compared to pure g-C_3_N_4_ (1.4%). The following work of this group (Wang et al., 2020a) devoted to further improving the selectivity for CH_4_ generation with high apparent quantum efficiency (AQE) under visible light irradiation, in which 3D ordered macroporous (3DOM) TiO_2_@C was coupled with g-C_3_N_4_ using Pt as electron mediator (3DOM-CNPTC) (shown in Figure 5D,E). The DFT calculation revealed the enrichment of photogenerated electorns by abundant N-sites on the interface between Pt and g-C_3_N_4_, which can reduce the adsorbed CO_2_ to CH_4_ directly in the presence of H_2_O, thereby improving the selectivity for CH_4_ generation (81.7%). In addition, the strong visible light adsorption by g-C_3_N_4_ and Pt as well as the multiple scattering of incident light within the 3DOM structure (Figure 5F) result in the high AQE of the Z-scheme heterojunction (5.67%), which is 140 folds than that of P25 (0.04%). Interestingly, the interaction between TiO_2_ and g-C_3_N_4_ could also be strengthened by Al-O links which was introduced into the Z-scheme through impregnation (Wu et al., 2019). Specifically, TiO_2_ nanotubes (TNTs) fabricated *via* anodization of Ti foils are dipped in AlCl_3_ solution followed by calcination to obtain Al-O-modified TNTs, which is then combined with porous g-C_3_N_4_ (PCN) *via* solid sublimation and transition of urea/NaHCO_3_ hybrid to from Al-O linked PCN/TNT composites. Results show that the low charge transfer efficiency at the interface between TiO_2_ and g-C_3_N_4_ caused by lattice mismatch of the two components can be significantly improved by introducing Al-O links to replace surface hydroxyl groups, thereby enhancing the separation efficiency of photogenerated charge carriers and benefiting for photoreduction of CO_2_ with increased yields of acetic acid, formic acid and methanol. According to Kuai’s research, rGO could also serve as electron mediator for the construction of Z-scheme heterojunction between TiO_2_ and CdS (Kuai et al., 2015). The remarkably prolonged photoluminasence (PL) decay time of CdS/rGO/TiO_2_ (2.4 ns) reveals the different electron migration mechanism compared to CdS/TiO_2_ (0.38 ns), which follows the carrier transfer mode in type II heterojunction. Obviously, the presence of rGO leads to the establishment of Z-scheme system, in which photogenerated electrons in the CB of TiO_2_ are extracted by rGO and then transferred to the VB of CdS to recombine with photogenerated holes there, resulting in the enrichment of photogenerated electrons and holes in the CB of CdS and the VB of TiO_2_, respectively. Although the photoreduction efficiency of CO_2_ is still low on CdS/rGO/TiO_2_, the attempt to construct Z-scheme heterojunction with low-cost carbon material instead of noble metal as electron mediator is successful, while the high selectivity for CH_4_ generation is also promising.

**FIGURE 6:**
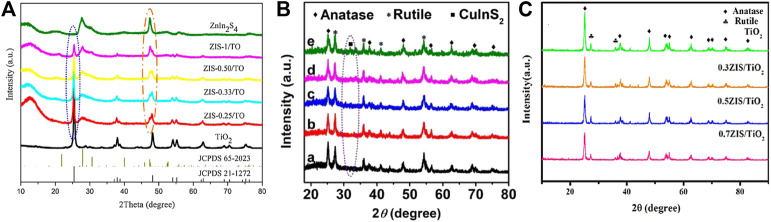
XRD patterns of **(A)** ZnInS_2_/TiO_2_ series, **(B)** CuInS_2_/TiO_2_ series and **(C)** Zn_3_In_2_S_6_/TiO_2_ series. Reproduced from Yang et al. (2017) and Xu et al. (2018) with permission from Elsevier and Copyright Clearance Center, and She et al. (2018) with permission from Wiley-VCH, respectively.

Recently, construction of direct Z-scheme system by coupling two different semiconductors with matching geometric and band structure has become research hotspot in the field of photocatalytic CO_2_ reduction, which is more facile to synthesis and more convenient for charge transfer at the interface. In particular, semiconductors with narrow band gap are more preferred in the TiO_2_-based direct Z-scheme heterojunction to improve the utilization of visible light. Typically, ZnInS_2_ nanosheets were decorated onto the surface of 1D TiO_2_ nanobelts *via* hydrothermal process (Yang et al., 2017). The authors claimed the Z-scheme electron transfer mechanism between ZnInS_2_ and TiO_2_ based on the experimental results of CH_4_ generation. Since the CB potential of TiO_2_ is lower than the redox potential of CO_2_/CH_4_, it is reasonable to believe that photogenerated electrons in the CB of ZnInS_2_ are retained due to the Z-scheme electron transfer mechanism and take in charge for photocatalytic CO_2_ reduction to produce CH_4_. However, stronger evidence is needed to prove this conjecture. In another work, a similar 3D hierarchical nanostructure was constructed by depositing CuInS_2_ nanoplates on TiO_2_ nanofibers (Xu et al., 2018b). DFT calculations revealed the higher Fermi level (E_F_) of CuInS_2_ than that of TiO_2_, which forces electrons transfer from CuInS_2_ to TiO_2_ after their contact and creates a build in internal electric filed at the interface. The recombination of photogenerated electrons in the CB of TiO_2_ and photogenerated holes in the VB of CuInS_2_ under the guidance of the internal electric filed leads to the accomplishment of high efficient Z-scheme charge transfer pathway. As a result, photogenerated electrons enriched in the CB of CuInS_2_ facilitate the photocatalytic reduction of CO_2_ to produce CH_4_ and CH_3_OH in the presence of protons provided by water oxidation. *In situ* irradiated X-ray photoelectron spectroscopy (ISI-XPS) was also applied to provide direct evidence of Z-scheme electron transfer mechanism (Low et al., 2019). The binding energy shifts of Ti 2p (by 0.3 eV) and Cd 3 days (by -0.2 eV) under light irradiation indicate the decreased electron density of TiO_2_ as well as the increased electron density of CdS, suggesting that photogenerated electrons migrates from TiO_2_ to CdS, which agrees well with Z-scheme mechanism. The ternary semiconductor of Zn_3_In_2_S_6_ was also used by She et al. for the construction of direct Z-scheme heterojunction with TiO_2_ (She et al., 2018). Higher CO and CH_4_ yields were realized on Zn_3_In_2_S_6_/TiO_2_ in comparison with ZnInS_2_/TiO_2_ and CuInS_2_/TiO_2_, which could be attributed to the higher crystallinity of the two constituents that favored for charge separation (shown in Figure 6). In addition, modification on TiO_2_ to narrow its band gap for the improved visible light adsorption is also an efficient strategy to further enhance the photocatalytic performance of the TiO_2_-based Z-scheme heterojunction. As reported by Truc et al., *E*_g_ of TiO_2_ (3.2 eV) was reduced to 2.91 eV after Nb doping (Thi Thanh Truc et al., 2019). The as-obtained Nb-TiO_2_ was grinded with melamine followed by calcination at 550 °C to form Nb-TiO_2_/g-C_3_N_4_ heterojunction with a clear boundary at the interface. The well matched lattice spacing of the TiO_2_ {101} (0.353 nm) and g-C_3_N_4_ {002} (0.320 nm) facets benefits to the electron transfer at the interface following Z-scheme mechanism, resulting in high efficiency for photocatalytic CO_2_ reduction. The advantages of low cost, full visible light spectrum responsibility (400–700 nm) and superior generation rate of CH_4_ (562 μmol g_cat_^−1^ h^−1^), CO (420 μmol g_cat_^−1^ h^−1^) and HCOOH (698 μmol g_cat_^−1^ h^−1^), make Nb-TiO_2_/g-C_3_N_4_ a promising VLD photocatalyst for practical application in the future to reduce the CO_2_ level in the atmosphere. Moreover, the high O_2_ yield of Nb-TiO_2_/g-C_3_N_4_ (1702 μmol g_cat_^−1^ h^−1^) indicates that the artificial Z-scheme system can mimic the nature photosynthesis by green plants well.

For a long time, stability is one of the main defects facing the photocatalysts that restricts their long-term performance. The photocatalytic activity decreased continuously in the process of illumination due to photocorrosion. Construction of Z-scheme heterojunction can also protect the narrow band gap semiconductor coupled with TiO_2_ from photo-oxidation. As reported by Aguirre et al., XPS spectra of Cu_2_O exhibited an increased Cu(II) content with the extension of illumination time, indicating that Cu(I) was partially oxidized by photogenerated holes (Aguirre et al., 2017). On the contrary, Cu(I) in the Cu_2_O/TiO_2_ heterojunction showed no obvious change in valence state, revealing the protection of TiO_2_ toward Cu_2_O by injecting photogenerated electrons into the VB of Cu_2_O to recombine with photogenerated holes there, which also demonstrated the Z-scheme electron transfer mechanism between TiO_2_ and Cu_2_O. Interestingly, a stable direct Z-scheme heterojunction can also be formed between TiO_2_ and metal organic frameworks (MOFs) as PCN-224(Cu) (Wang L. et al., 2019). It is worth nothing that the high specific surface area as well as porous structure of MOFs benefits for CO_2_ adsorption, while the alternative ligands endow MOFs with adjustable band structure and spectra response range, thus providing a series of promising candidates for the design and construction of direct Z-scheme systems for efficient photocatalytic CO_2_ reduction. With deepening of the research on the Z-scheme photocatalytic system and the recognition of its photocatalytic performance, more and more different types of Z-scheme photocatalysts have been developed (Raza et al., 2020; Zeng et al., 2020), accelerating the process of photocatalytic CO_2_ reduction from basic research to practical application.

### TiO_2_ Based S-M Heterojunction for CO_2_ Photoreduction

**FIGURE 7:**
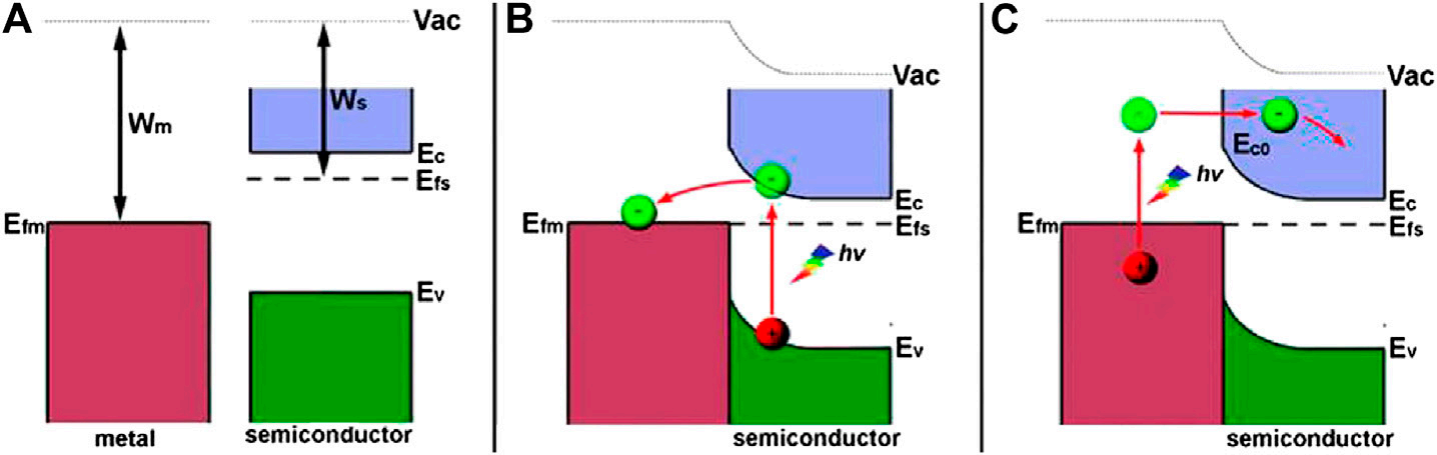
Band structure of semiconductor **(A)** before contact and **(B)** after contact with metal to form the Schottky barrier; **(C)** the SPR effect induced hot electron transfer. Reproduced from Bai et al. (2015) with permission from the Royal Society of Chemistry.

As reported by previous literatures, TiO_2_ modified by metal nanoparticles exhibits enhanced photocatalytic performance due to the promoted charge carrier separation efficiency, expanded light adsorption range as well as high selectivity toward reduction products. In general, Schottky barrier at the interface of semiconductor and metal prevents recombination of photogenerated *e* ^−^/*h* ^+^ pairs (Ma et al., 2014; Wang et al., 2014; Ola and Maroto-Valer, 2015; Li et al., 2019). Specifically, the higher work function of metal (W_m_) than that of semiconductor (W_s_) results in the higher Fermi level of semiconductor (E_Fs_) than that of metal (E_Fm_) (shown in Figure 7A). Contacting semiconductor with metal leads to charge transfer at the interface until the alignment of their Fermi levels. During the process, migration of electrons from semiconductor to metal results in band bending of the semiconductor and creates a space charge region at the interface (Schottky barrier), which could prevent backflow of photogenerated electrons to inhibit their recombination with photogenerated holes (shown in Figure 7B). The promoted charge separation efficiency benefits for photocatalytic CO_2_ reduction as well as the enhanced water oxidation efficiency. In addition, metals can enrich electrons to create high electron density regions on their surfaces, which are favoring for photoreducing CO_2_ to hydrocarbons in the presence of water due to their lower redox potential than CO. Besides, local surface plasmon resonance (LSPR) effect of certain metals can enhance visible light adsorption of the Schottky heterojunction and inject hot electrons into the CB of semiconductor, thereby facilitating the photoreduction of CO_2_ (shown in Figure 7C). In this section, strategies for coupling TiO_2_ with different metal nanoparticles as well as their different enhancement mechanisms of photocatalytic performance will be reviewed and discussed in detail. Photocatalytic CO_2_ reduction performance of the typical TiO_2_-based S-M heterojunctions are listed in Table 3.

**FIGURE 8:**
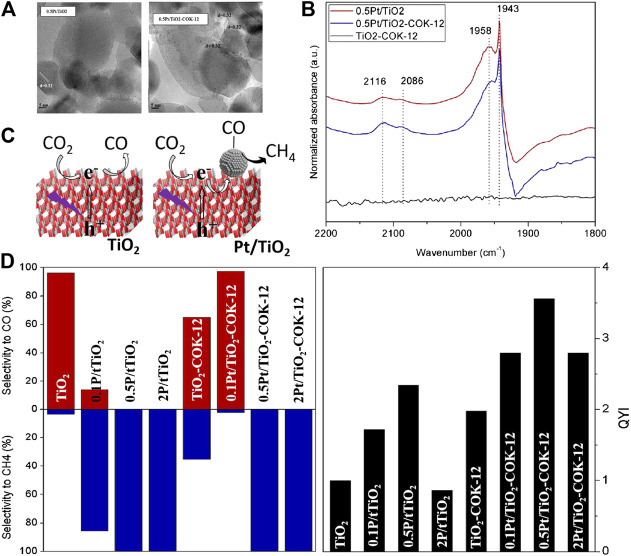
**(A)** TEM images of 0.5 Pt/TiO_2_ and 0.5 Pt/TiO_2_-COK-12, **(B)** FTIR spectra of the TiO_2_, 0.5 Pt/TiO_2_ and 0.5 Pt/TiO_2_-COK-12 catalysts after reaction, in the CO stretching region, **(C)** Schematic representation of the proposed reaction pathway over TiO_2_ and Pt/TiO_2_ catalysts, and **(D)** Net selectivities and quantum yield indices (QYI) obtained with the different catalysts. The QYI with TiO_2_ is 1 by definition. Reproduced from Tasbihi et al. (2018a) with permission from Elsevier and Copyright Clearance Center.

As a classic noble metal cocatalyst, Pt has been widely used in the photocatalysis field for both water splitting and CO_2_ reduction. In particular, TiO_2_ loaded with Pt nanoparticles has been demonstrated to be efficient for photoreduction CO_2_ to CH_4_. As reported by Fresno et al., a series of Pt/TiO_2_ photocatalysts with different Pt loading amount were fabricated by treating P25 in the Pt precursor-contained aqueous solution *via* the deposition-precipitation procedure (Tasbihi et al., 2018a). The as-obtained sample (Figure 8A) displays ca. 100% selectivity toward CH_4_ generation at the optimum Pt content (>0.58 wt% of TiO_2_), which can be ascribed to the strong chemisorption of CO on Pt nanoparticles (proved by the FTIR spectra in ATR mode (shown in Figure 8B) along with *in-situ* NAP-XPS analysis) and the further reduction of CO to CH_4_ (shown in Figure 8C). However, the adsorption features of CO can not be observed on bare TiO_2_, which yields CO as the main product under the same condition. The correspondingly net selectivities and quantum yield indices (QYI) are shown in Figure 8D. In another work, Pt/TiO_2_ was synthesized by the hydrolysis of Titanium (IV) butoxide (TEOT) in the presence of H_2_PtCl_6_∙6H_2_O, resulting in the doping of Pt^2+^ into the lattice of TiO_2_ and loading of Pt nanoparicles (Pt^0^) on the surface of TiO_2_ (Xiong et al., 2015). The low recombination efficiency of photogenetated *e* ^−^/*h* ^+^ pairs due to the deposition of Pt^0^ as well as strong visible light adsorption attributed to Pt^2+^ doping significantly enhance photocatalytic performance of Pt^2+^-Pt^0^/TiO_2_ with higher quantum yield (1.42%) for CO_2_ conversion than that of bare TiO_2_ (0.36%). Moreover, plenty of electrons enriched by Pt^0^ and protons supplied by water oxidation benefit for the high selectivity toward CH_4_ formation (E_red_/SCE = −0.48 V). Compared with the formation of CO (E_red_/SCE = -0.77 V), this reaction is more feasible in thermodynamics. In summary, the kinetic feasibility (strong chemisorption of CO on Pt) and thermodynamic convenience contribute to photoreduction of CO_2_ to CH_4_. On this basis, Pt/TiO_2_ were deposited on porous supports with large surface area (etc. COK-12 (Tasbihi et al., 2018a) and Al_2_O_3_ foam (Tasbihi et al., 2018b)) to promote active sites exposure and achieve higher CH_4_ yield.

**FIGURE 9:**
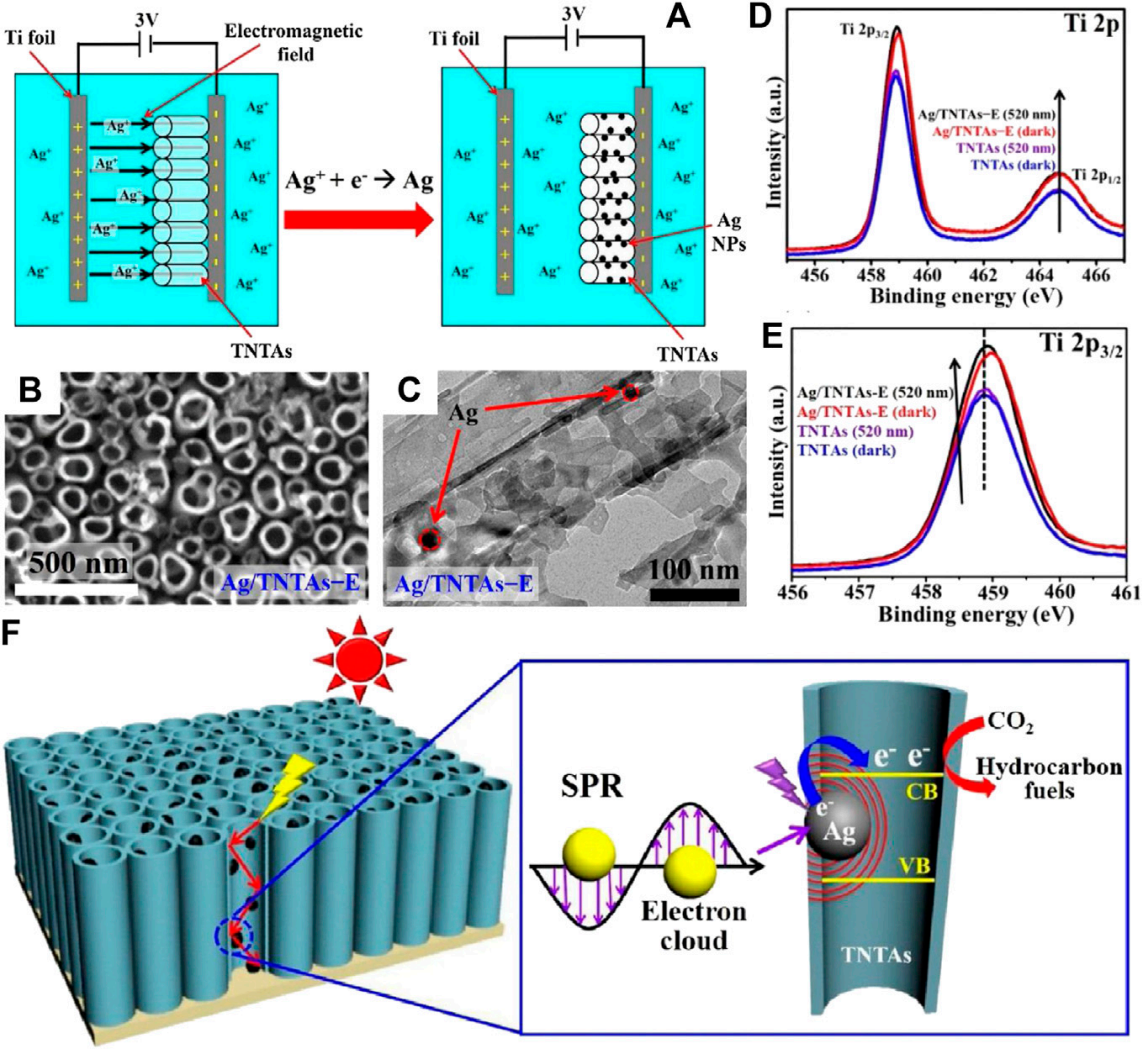
**(A)** Schematic illustration of electrochemical deposition methods for loading Ag NPs into the TNTAs, **(B)** SEM image (top view) and **(C)** TEM image (side view) of Ag/TNTAs-E, Comparison of high-resolution SIXPS spectra of **(D)** Ti 2p and **(E)** Ti 2p_3/2_ for TNTAs and Ag/TNTAs-E in the dark and under 520 nm LED light irradiation, and **(F)** Schematic illustration of the enhanced SPR effect of Ag NPs in the TNTAs structure. Reproduced from Low et al. (2018) with permission from Elsevier and Copyright Clearance Center.

Coupling TiO_2_ with noble metals that can induce the LSPR effect has also been adapted by researchers for efficient photocatalytic CO_2_ reduction. As reported by Wang et al., 0D/2D Au/TiO_2_ was synthesized by *in situ* growth of Au nanoparticles on the surface of TiO_2_ nanosheets *via* chemically reduction (Wang R. et al., 2019). The hot electrons induced by the LSPR effect of Au under visible light irradiation (550 nm) could inject into the CB of TiO_2_ and reduce CO_2_ to CO. However, as the only electron source (TiO_2_ can not be excited by visible light), the limited hot electrons cannot further reduce CO to CH_4_. Interestingly, the Au/TiO_2_ hybrid yielded CH_4_ as the main product under 300 W Xe lamp irradiation that contained a certain amount of UV light. Specifically, recombination of photogenerated electrons and holes was suppressed owing to the Schottky barrier that facilitated transfer of *e* ^−^ from the CB of TiO_2_ to Au. Moreover, *h* ^+^ remained in the VB of TiO_2_ could oxidize water to provided plenty of protons for CH_4_ generation. On this basis, facet engineering was introduced by Wang et al. for the rational design of interface between Au and exposed facet of TiO_2_ to realize higher charge separation efficiency (Wang et al., 2021). Results showed that the lower height of Schottky barrier on the Au/TiO_2_{101} interface resulted in more smooth migration of photogenerated electrons from the CB of TiO_2_ to Au compared to the Au/TiO_2_{001} interface, thereby exhibiting better performance for photocatalytic CO_2_ reduction. Notably, the architecture of TiO_2_ among Au/TiO_2_ heterojunction also plays an important role for efficient photoreduction of CO_2_ (Jiao et al., 2015; Khatun et al., 2019). Jiao et al. (Jiao et al., 2015) prepared 3D ordered macroporous (3DOM) TiO_2_ to support Au nanopaticles, which were uniformly dispersed in the inner wall of the 3DOM structure. The multiple scattering of incident light within the 3DOM structure enhanced light utilization efficiency of the heterojunction, while the ordered macroporous also improved the mass transfer efficiency of the reactants. In addition, the SPR effect of Au induced by visible light irradiation provided extra electrons for photocatalytic CO_2_ reduction, which was benefited for CH_4_ generation. In another work, electrochemical anodization was used to fabricate TiO_2_ nanotubes (TNTs) with light adsorption edge in the visible region, indicating its weak photocatalytic activity illuminated by visible light (Khatun et al., 2019). Coupling with Au by electronchemical deposition significantly improved visible light adsorption of TNTs and promoted charge separation efficiency due to the LSPR effect of Au nanoparticles. The excellent CH_4_ yield (14.67% of CO_2_ was converted to CH_4_) under visible light irradiation made Au-TNTs a very promising solar-driven photocatalyst to convert CO_2_ into hydrocarbon fuels. Similarly, plasmonic Ag were electronchemical deposited into the inner space of TiO_2_ nanotube arrays (Figure 9A) to investigate the enhancement of SPR effect on photocatalytic performance, while the morphology and structure of as-obtained Ag-TNTAs-E are shown in Figures 9B,C (Low et al., 2018). The direct evidences of the existence of Schottky barrier between Ag and TiO_2_ as well as migration of hot electrons induced by the SPR effect of Ag nanoparticles can be found in the synchronous-illumination X-ray photoelectron spectroscopy (SIXPS) spectra based on the shift of Ti 2p_3/2_ peak before and after illumination (Figures 9D,E). Moreover, the SPR effect of Ag nanoparticles was strengthened by the multiple scatted light in TNTAs (Figure 9F), while the derived near field effect accelerated charge transfer at the heterointerface to promote separation efficiency of photogenerated *e* ^−^/*h* ^+^ pairs, thereby endowing enhanced VLD activity of Ag-TNTAs for CO_2_ photoreduction. Although promoting photoreduction efficiency of CO_2_ and clarifying the involved mechanisms are the focus of current research, the improvement of photocatalyst synthesis methods also deserves attention. The silver mirror reaction was adopted by Yu et al. to deposited Ag on TiO_2_ nanoparticles (Yu et al., 2016). CH_3_OH generated in CO_2_-saturated 1 M NaHCO_3_ solution is the main product of photocatalytic CO_2_ reduction, which is more valuable than the primary products such as CO and CH_4_. In another work, Ag(I) adsorbed by TiO_2_ nanorod arrays were completely reduced by cold plasma within 30 s to form uniformly distributed Ag nanoparticles (Cheng et al., 2017). This fast and efficient strategy is very promising for the fabrication of metal nanoparticles decorated semiconductor photocatalysts on a large scale.

Bimetallic nanoalloys that combined advantages of the two metal components are efficient cocatalysts for photocatalytic CO_2_ reduction and have been introduced in the TiO_2_-based photocatalytic systems. As reported by Neaţu et al., Au and Cu species were deposited on TiO_2_ nanopartcles stepwisely followed by calcining in H_2_ atmosphere to form Au-Cu alloy (Neaţu et al., 2014). In this case, Au is served as visible light harvester due to its LSPR effect while Cu can covalently bind with CO reduced from CO_2_ and direct the generation of CH_4_. Therefore, high VLD photocatalytic activity with outstanding CH_4_ selectivity (97%) was achieved. Other bimetallic nanoalloys, such as Au-Ag (Tahir et al., 2017), Au-Pd (Ziarati et al., 2020), Ag-Pd (Tan et al., 2018) and Pt-Ru (Wei Y. et al., 2018) nanoparticles are also been used to enhance photocatalytic performance of TiO_2_ for selective reduction of CO_2_. Among them, the combination of bimetallic nanoalloys and modified TiO_2_ (etc. hydrogenated black TiO_2_ (TiO_2-x_H_x_) (Ziarati et al., 2020) and N-doped TiO_2_ (Tan et al., 2018)) exhibited considerably enhanced visible light utilization and charge separation efficiency, which could become the future development trend of TiO_2_-based S-M heterojunction for solar-driven CO_2_ photoreduction. Moreover, construction of S-M heterojunction with hierachical architecture is also in great demand (Ziarati et al., 2020).

### TiO_2_ Based S-C Heterojunction for CO_2_ Photoreduction

Recently, coupling TiO_2_ with carbon-based nanomaterials including graphene and its derivatives (etc. graphene (GR) (Tu et al., 2013; Xiong et al., 2016; Biswas et al., 2018; Jung et al., 2018; Shehzad et al., 2018; Zhao et al., 2018; Zubair et al., 2018; Bie et al., 2019), graphene oxide (GO) (Chowdhury et al., 2015; Tan et al., 2017) and reduced graphene oxide (rGO) (An et al., 2014; Kuai et al., 2015; Sim et al., 2015; Tan et al., 2015; Lin et al., 2017; Olowoyo et al., 2019)), carbon nanotubes (CNTs) (Xia et al., 2007; Gui et al., 2014; Gui et al., 2015; Olowoyo et al., 2018; Rodríguez et al., 2020) and carbon quantum dots (CQDs) (Li et al., 2018; Wang K. et al., 2019) to construct TiO_2_-carbon heterojunction for photocatalytic reduction of CO_2_ has been widely concerned. The unique physicochemical properties of nanocarbon that responsible for the enhanced photocatalytic performance of the S-C heterojunction can be concluded as follows: 1) the large surface area and high mechanical stability of nanocarbon could provide a stable support for the uniformly distributed TiO_2_ nanoparticles with increased exposure of active sites and enhanced CO_2_ adsorption capacity; 2) the high charge carrier mobility, large capacitance of nanocarbon as well as the formation of Ti-O-C bond at the highly dispersed S-C interface facilitates the migration of electrons from TiO_2_ to carbon materials, thereby enhancing the separation efficiency of photogenerated *e* ^−^ and *h* ^+^ and inhibiting their recombination; 3) the optical properties of carbon materials, such as good optical transparency and wide spectrum adsorption range (especially for CQDs, expands to near IR region), contribute to the utilization of visible light of the TiO_2_-based S-C heterojunction and result in the improved quantum efficiency. In this section, S-C heterojunctions including TiO_2_-GR series, TiO_2_-CNT and TiO_2_-CQDs are reviewed and discussed in detail, respectively. Photocatalytic CO_2_ reduction performance of the typical TiO_2_-based S-C heterojunctions are listed in Table 3.

#### Coupling TiO_2_ With Graphene and Its Derivatives

Construction of TiO_2_-carbon heterojunction using graphene or its derivatives as the guest/host component derives improved photocatalytic performance due to its excellent electrical properties and chemical stability. It is worth nothing that the path of graphite-GO-rGO has been generally adopted by researchers to obtain graphene, whereas various of strategies have been developed for the fabrication of TiO_2_-graphene nanocomposites. As reported by Tu et al., *in situ* simultaneous reduction-hydrolysis technique was developed for the fabrication of TiO_2_-graphene 2D sandwich-like hybrid nanosheets (Tu et al., 2013). During the process, GO was reduced to graphene (rGO) by ethylenediamine (En) while Ti (IV) was hydrolyzed to TiO_2_ nanoparticles and loaded on rGO through Ti-O-C bonds. The abundant surface Ti^3+^ sites generated from En reduction could trap photogenerated electrons efficiently, thereby decreasing the recombination efficiency of charge carriers. Moreover, the synergism of Ti^3+^ sites and garphene favors for the generation of C_2_H_6_, which is inspiring for C-C coupling during the photoreduction process of CO_2_. In another work, the suspension of GO and TiO_2_ in ethanol was ultrasonicated and refluxed to form Ti-O-C bonds, while GO was partially reduced to rGO during the process (Shehzad et al., 2018a). The tightly connected two phases improve charge separation at the heterointerface, while the enlarged light absorption coefficient is attributed to the reduced bandgap energy by the formation the Ti-O-C bonds. As a result, the rGO/TiO_2_ nanocomposites exhibited greater yields of CH_4_ (12.75 μmol g_cat_^−1^ h^−1^) and CO (11.93 μmol g_cat_^−1^ h^−1^) than anatase for 4 folds. Theoretical calculation was applied by Olowoyo et al. to investigate the enhanced photocatalytic performance of rGO/TiO_2_ in reducing CO_2_ (Olowoyo et al., 2019). Results reveal that the high electron density of rGO has significant influence on the TiO_2_ bands and endows visible light responsibility of the composite. Moreover, the different electron migration paths within rGO/TiO_2_ under different light sources were observed. Compared to the electron transfer from TiO_2_ to rGO under UVA, irradiation by visible light leads to the direct generation of electorns and holes in rGO or TiO_2_, respectively. Both of the two pathways are efficient for photogenerated charge separation and favor for methanol production. In addition, the large adsorption capacity of CO_2_ is another feature of TiO_2_/graphene that contribute to photocatalytic CO_2_ reduction (Chowdhury et al., 2015). As reported by Chowdhury et al., TiO_2_/GO nanocomposites was obtained from the aqueous suspension of GO and TiO_2_ under ultrasonication flowed by continuous stirring. The synergism of physisorption (intermolecular electrostatic interactions as van der Waals forces or London dispersion forces) and chemisorption (coordination of O atoms with surface Lewis acid center (Ti sites), or coordination of C atom with surface Lewis acid center (oxygen functionalities of GO or TiO_2_)) led to the high adsorption amount of CO_2_ (1.88 mmol g^−1^), which facilitated its activation and photocatalytic reduction. Considering that photoreduction of CO_2_ requires the participation of electrons and protons produced by photooxidation of H_2_O, the adsorption capability of H_2_O molecule by photocatalyst is also critical. In view of the compete adsorption of H_2_O and CO_2_ on the active sites, proper partial pressures of CO_2_ and H_2_O are in great demand that determines the CH_4_ yield on GO-doped oxygen-rich TiO_2_ (Tan et al., 2017). Notably, the CO_2_ adsorption capacity and charge separation efficiency can be further improved by introducing nitrogen dopants into rGO to form NrGO/TiO_2_ system (Lin et al., 2017). On the one hand, the positive electrostatic potential regions created by nitrogen dopants on the surface of NrGO benefit for CO_2_ adsorption and activation. On the other hand, the injection of electrons from quaternary-N species existed in NrGO matrix to the delocalized π-system facilitates the transfer of electrons and inhibits the recombination of photogenerated *e* ^−^/*h* ^+^ pairs. These findings are very helpful for the design of photocatalytic system based on heteroatom-doped graphene for CO_2_ conversion with high efficiency. Furthermore, construction of well-defined nanostructure offers another thought to improve photocatalytic performance of TiO_2_/graphene heterojunction (Zubair et al., 2018). Typically, TiO_2_ nanotube arrays (TNT) with attractive 1D vectorial charge transfer can suppress recombination of photogenerated charge carriers efficiently. Graphene quantum dots (GQDs) decorated on TiO_2_ nanotube (G-TNT) promotes charge transfer at the heterointerface, which could also enhance the light utilization of the heterojunction due to its superior visible light responsibility. Besides, the high surface area of the heterojunction contributes to the exposure of active sites that favor for CO_2_ adsorption and activation. Consequently, a 5.6 fold CH_4_ yield was obtained on G-TNT in comparison with pure TNT. On this basis, Pt nanoparticles were employed for the construction of rGO/Pt-TNT ternary composite with promoted visible light responsibility and photoreduction selectivity of CO_2_ to produce CH_4_ (Sim et al., 2015). In particular, the LSPR effect of Pt expands light adsorption range of the heterojunction to 450 nm that can be activated by visible light. Similarly, the high selectivity for CH_4_ generation (99.1%, compared to CO yield) was also proved by Zhao et al. on (Pt/TiO_2_)@rGO system (Zhao H. et al., 2018), in which vectorial electron transfer from TiO_2_ to rGO through Pt was also demonstrated.

#### Coupling TiO_2_ With CNT

Generally, charge transfer along the 1D CNT leads to high separation efficiency of photogenerated *e* ^−^/*h* ^+^ pairs and endows superior photocatalytic performance of TiO_2_/CNT heterojunction. Moreover, CNT can also serve as support to reduce the aggregation of TiO_2_ nanoparticles, thus resulting in the formation of highly dispersed heterointerface and large exposure of active sites. As reported by Xia et al., the multi-walled CNT (MWCNT)/TiO_2_ hybrid fabricated *via* sol-gel method yielded C_2_H_5_OH as the main photoreduction product of CO_2_ under UV light irradiation (Xia et al., 2007). In a further study, MWCNT/TiO_2_ with core-shell nanostructure was demonstrated to be visible light active (due to the excellent visible light adsorption ability of CNT) that can convert CO_2_ to CH_4_ (Gui et al., 2014). A following work carried out by this group introduced Ag nanoparticles to MWCNT/TiO_2_ system (Ag-MWCNT@TiO_2_) for the further enhanced photocatalytic performance (Gui et al., 2015). In particular, the Schottky barrier between Ag and TiO_2_ prevents backflow of photogenerated electrons that transferred from TiO_2_ to Ag. The synergism of MWCNT and Ag nanoparticles greatly enhances separation efficiency of photogenerated charge carriers and restricts their recombination, thereby resulting in higher CH_4_ formation rate (0.91 μmol g_cat_^−1^ h^−1^) in comparison with the binary system (MWCNT@TiO_2_, 0.17 μmol g_cat_^−1^ h^−1^). Further studies revealed the mechanism of electron transfer between TiO_2_ and CNT (Olowoyo et al., 2019). Specifically, strong attachment between MWCNT and the {101} facet of anatase TiO_2_ introduces common orbitals within the band gap of TiO_2_, which is the fundamental of charge transfer between the two phases and enables visible light responsibility of the composites. Electron transfer from TiO_2_ with higher density of initial states to MWCNT under irradiation of both UVA and visible light is more probable basing on the computation results. In addition, the tight contact between MWCNT and TiO_2_ due to the combination of sonothermal and hydrothermal methods results in extremely high photocatalytic activity with CH_3_OH generation rate of 2360 μmol g_cat_^−1^ h^−1^, much higher than that of the similar photocatalytic systems. A recent study reported the fabrication of TiO_2_/CNT composite in the medium of supercritical CO_2_ (Rodríguez et al., 2020). Although photocatalytic activity of the product is relatively weak (CO, 8.1 μmol g_cat_^−1^ h^−1^; CH_4_, 1.1 μmol g_cat_^−1^ h^−1^), it can also provide a novel strategy for the synthesis of TiO_2_-based photocatalyst and leave a large space for performance improvement.

#### Coupling TiO_2_ With CQDs

As a new class of 0D carbon-based nanomaterial, the excellent photoelectric properties of CQDs, such as wide spectral response range, photo-induced charge transfer ability, up-conversion function and anti-photocorrosion property, make it a promising cocatalyst to enhance photocatalytic performance of traditional semiconductor photocatalyst as TiO_2_ (Li et al., 2018; Wang K. et al., 2019). As reported by Li et al., N, S-containing CQDs (NCQDs) was synthesized *via* a microwave-assisted method using thiourea and citric acid as precursors, which then assembled with P25 under continuous stirring at 80°C to form NCQDs-TiO_2_ nanocomposites. Particularly, TiO_2_ sensitized by NCQDs can be activated by visible light, while electrons transferred from rutile TiO_2_ in P25 to NCQDs prevented recombination of photogenerated *e* ^−^/*h* ^+^ pairs, thus enhancing photoreduction efficiency of CO_2_. Although the photoelectric properties of CQDs are attractive, relative research about coupling CQDs with TiO_2_ or other semiconductors to construct heterostructured photocatalyst for photocatalytic CO_2_ reduction is still few. In our opinion, as an important characteristic distinguished from other carbon-based materials, up-conversion function of CQDs is worth developing to improve the light energy utilization of heterostructured photocatalysts, especially for the wide band gap semiconductor-contained systems.

**FIGURE 10:**
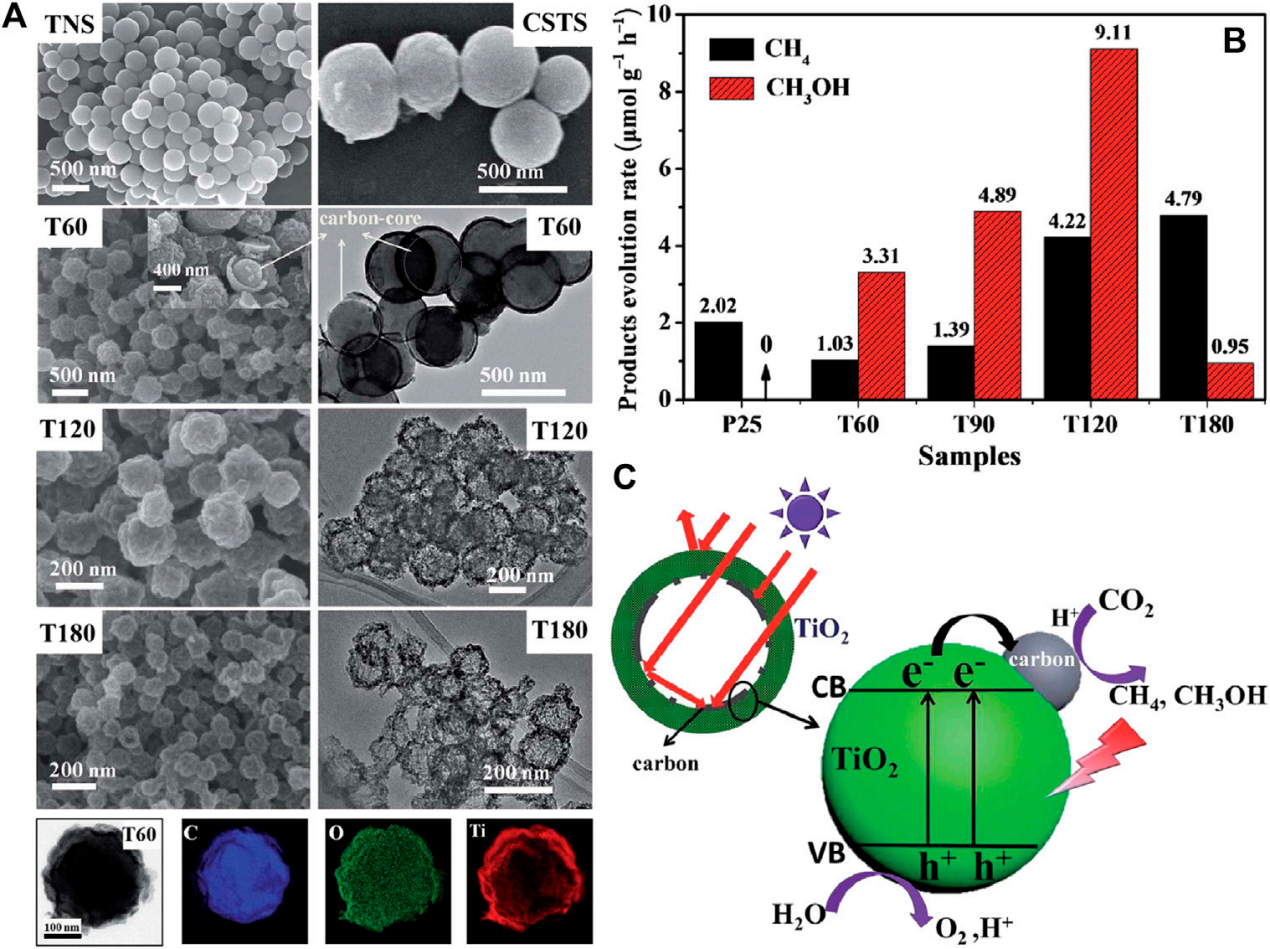
**(A)** FESEM images of TNS, CSTS, T60, T120 and T180, and TEM images of T60, T120 and T180, and STEM image of T60 and the corresponding elemental mapping images of C, O and Ti; **(B)** Comparison of the photocatalytic CH_4_ or CH_3_OH evolution rate of carbon@TiO_2_ composite samples and P25 (under simulated solar light); and **(C)** Photoexcitation process of the carbon@TiO_2_ composite photocatalyst with hollow structure. Reproduced from Wang et al. (2017) with permission from the Royal Society of Chemistry.

In addition to carbon-based nanomaterials analyzed above, other carbon forms can also combine with TiO_2_ to obtain heterojunction for photocatalytic CO_2_ reduction with high efficiency. For instance, Zhang et al. coated TiO_2_ on electrospun carbon nanofibers to promote active sites exposure as well as charge separation and transfer of the nanocomposites (Zhang J. et al., 2018). Besides, the heat produced by carbon nanofibers due to its photothermal conversion function accelerates the diffusion kinetics of reactants and products during photocatalytic process, thus further enhancing the photoreduction efficiency of CO_2_. The local photothermal effect induced by carbon species is also highlighted by Wang et al. among the hybrid carbon@TiO_2_ hollow spheres (Wang et al., 2017). Moreover, the multiple scattering of incident light within the hollow structure improves light utilization of the hybrid (shown in Figure 10) and contributes to improve the quantum efficiency of photocatalytic CO_2_ reduction. This result indicates that not only component, but also architecture of the heterojunction photocatalysts plays important role in improving photocatalytic performance.

### TiO_2_ Based Multicomponent Heterojunction for CO_2_ Photoreductions

**FIGURE 11:**
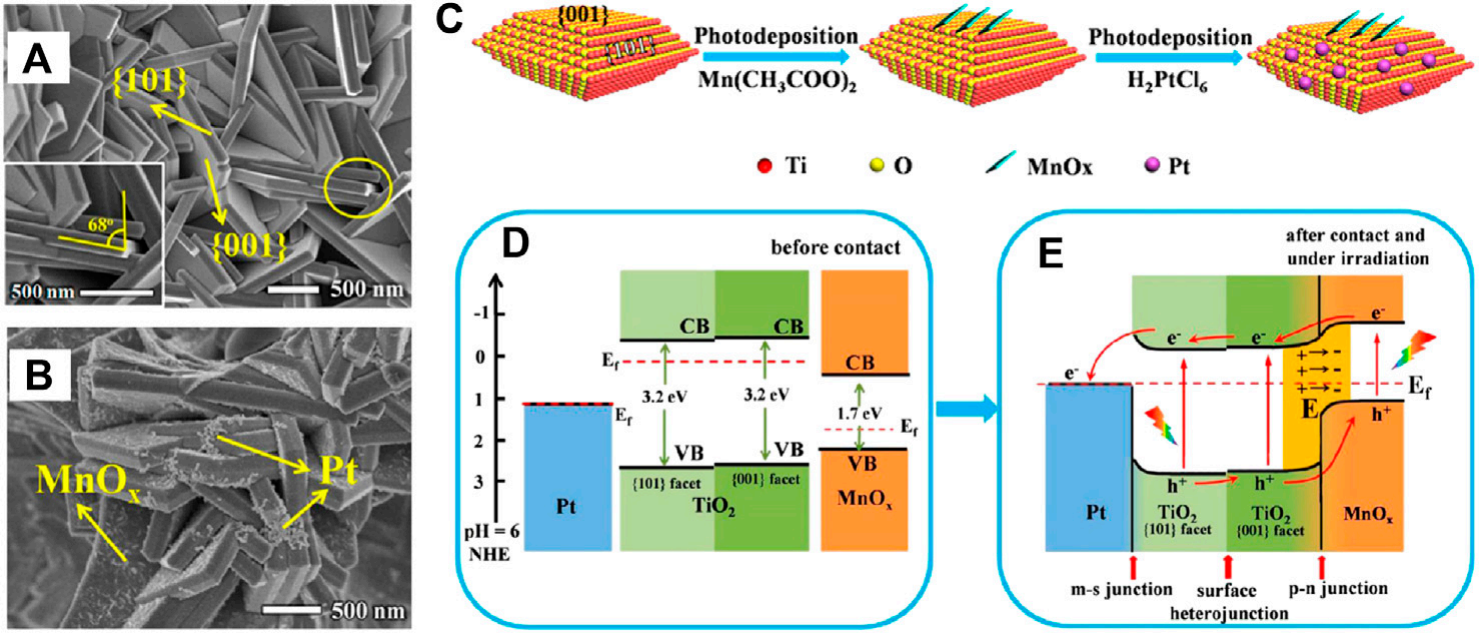
SEM images of as-prepared **(A)** TiO_2_ and **(B)** TiO_2_-MnO_x_-Pt (TMP); **(C)** Schematic diagram of selective photodeposition process of MnO_x_ nanoflakes and Pt nanoparticles on anatase TiO_2_ {001} and {101} facets; Schematic diagram of proposed photocatalytic CO_2_ reduction mechanism of sample TMP. The relative band energy positions of TiO_2_, Pt, and MnO_x_ **(A)** before contact and **(B)** after contact and under irradiation. Reproduced from Meng et al. (2019) with permission from the American Chemical Society.

Construction of TiO_2_-based multicomponent heterojunction to introduce two different functional co-catalysts for efficient VLD photocatalytic CO_2_ reduction has been widely adopted, in which TiO_2_ combined with any two of another semiconductor (AS), metal nanoparticles (MNPs) and nanocarbon (C) to form ternary composites is currently the most studied system. Previous research revealed the highest selectivity of Pt for CH_4_ generation compared to other noble metal cocatalysts (Pt > Pd > Au > Rh > Ag) due to its excellent electron extraction ability that derives high electron density around it and facilitates CO_2_ photoreduction (Xie et al., 2014). However, the consequent increase in H_2_ production is unfavorable and should be suppressed to realize further enhanced photoreduction efficiency of CO_2_. Xie et al. coated MgO amorphous layers on Pt/TiO_2_ hybrid to improve chemisorption of CO_2_, which was then reduced to CH_4_ directly by photogenerated electrons enriched on adjacent Pt nanoparticles with high efficiency, thus benefiting for the selective formation of CH_4_. The similar function of Cu_2_O was demonstrated by Xiong et al. from the Pt-Cu_2_O/TiO_2_ nanocomposite (Xiong et al., 2017c). Notably, the charge separation efficiency of the ternary system decreased with the increasing amount of MgO, indicating that excess MgO may restrict electrons transfer from TiO_2_ to Pt. Therefore, TiO_2_/MNPs/AS ternary heterojunctions with rational designed architecture and efficient carrier migration path are necessary. According to Meng’s research, MnO_x_ and Pt were selectively deposited on the {001} and {101} facet of TiO_2_ (Figure 11A,B,C), respectively (Meng et al., 2019). The series connection of S-M (Pt and TiO_2_{101}), facet (TiO_2_{101} and {001}) and p-n (TiO_2_{101} and MnO_x_) heterojunction accelerated migration of photogenerated electrons along the path of MnOx→TiO_2_{001}→TiO_2_{101}→Pt while photogenerated holes in the opposite direction (shown in Figure 11D,F). As a result, the separation efficiency of photogenerated charge carriers is greatly improved, so as to the enhanced photocatalytic performance with CH_4_ and CH_3_OH as the main products. In another work, Z-scheme heterojunction that is favoring for the recombination of inefficient charge carriers was constructed by coupling TiO_2_ and ZnFe_2_O_4_ using Ag as electron mediator (Tahir, 2020). Superior CO yield (1025 μmol g_cat_^−1^ h^−1^) accompanied with the generation of CH_4_ (132 μmol g_cat_^−1^ h^−1^) and CH_3_OH (31 μmol g_cat_^−1^ h^−1^) should be attributed to the enhanced charge separation efficiency under UV light irradiation. It is worth nothing that the magnetic properties of ZnFe_2_O_4_ should not be ignored which facilitate the recovery of photocatalyst from solid-liquid suspension, although the solid-gas mode is undertaken in this study. Moreover, Ag could also promote visible light adsorption of the ternary system due to its strong LSPR effect, which had been demonstrated by Xu et al. using MgO-Ag-TiO_2_ as photocatalyst (Xu and Carter, 2019). In order to further investigate the synergism of the LSPR effect and chemisorption of CO_2_ on the improvement of photoreduction efficiency of CO_2_, TiO_2_/MNPs/AS heterojunctions as Au/Al_2_O_3_/TiO_2_ (Zhao Y. et al., 2018) and Au@TiO_2_ hollow spheres (THS)@CoO (Zhu et al., 2019) were synthesized. On this basis, MgAl layered double oxides (MgAl-LDO) were developed to provide both Lewis basic sites (MgO) and Lewis acid sites (Al_2_O_3_) for CO_2_ chemisorption and H_2_O dissociation among the Pt/MgAl-LDO/TiO_2_ nanocomposite, respectively (Chong et al., 2018). Benefiting from the generation of monodentate carbonate (m-CO_3_^2-^) on the surface of MgO accompanied with the supply of H^+^ from H_2_O dissociation by Al_2_O_3_, the activation of CO_2_ became easier and led to the increased CH_4_ yield by 11 folds compared to Pt/TiO_2_.

In a typical TiO_2_/C/AS system, nanocarbon is served as electron channel to guide photogenerated electrons flow from TiO_2_ to the AS while photogenerated holes left in the VB of TiO_2_, thereby resulting in the spatial separation of photoinduced redox reactions with enhanced CO_2_ photoreduction efficiency. As reported by Jung et al., mesoporous TiO_2_ and a few layers of MoS_2_ were assembled with graphene aerogel *via* one-pot hydrothermal method to construct a 3D hierarchical structure (Jung et al., 2018). In addition to high separation efficiency of photogenerated *e* ^−^/*h* ^+^ pairs, the improvement of light utilization and mass transfer efficiency based on the 3D structure of graphene with efficient visible light adsorption (Biswas et al., 2018) is also important factor that contribute to photocatalytic CO_2_ reduction. Compared to semiconductors as MoS_2_ (Jung et al., 2018), CuGaS_2_ (Takayama et al., 2017) or WSe_2_ (Biswas et al., 2018), noble metal nanoparticles are more efficient for the accumulation of photogenerated electrons transferred through electron channel (graphene) and favoring for the generation of more valuable products as CH_4_ and CH_3_OH (Tan et al., 2015; Meng et al., 2019; Tahir, 2020). However, the high cost as well as relative low resistance toward photocorrosion restricts their application on a large scale. Photocatalytic CO_2_ reduction performance of the typical TiO_2_-based multicomponent heterojunctions above are listed in Table 3. In the future, the development of noble metal-free multicomponent heterojunction based on TiO_2_ for efficient solar-driven photocatalytic CO_2_ reduction will become one of the main directions in this field.

### TiO_2_ Based Phase and Facet Heterojunctions for CO_2_ Photoreduction

#### TiO_2_ Based Phase Heterojunction

Phase heterojunction composed of different crystal phases of the same semiconductor exhibits greater photocatalytic activity than the single-phased photocatalyst (Ma et al., 2014; Wei L. et al., 2018; Nguyen et al., 2020). This is because the contact of crystal phases with different energy band structure leads to an increase in carrier separation efficiency at the interface. Moreover, the unique interfacial trapping sites may become new photocatalytic active sites. As is known, there are four main crystal phases of TiO_2_ (including anatase, rutile, brookite, and TiO_2_ (B)) existed in nature, in which rutile is the most thermodynamically stable phase and can be obtained by calcining the other three polymorphs (Ma et al., 2014). Among them, as the two most widely studied TiO_2_ crystal phases with photocatalytic activity, the difference in the lattice structure of anatase and rutile leads to different electronic band structures, and ultimately results in a difference in band gap width. Compared to rutile (*E*_g_ = 3.02 eV for bulk material), anatase tends to show higher photocatalytic activity due to its wider band gap (*E*_g_ = 3.20 eV for bulk material) that gives stronger redox ability to the photogenerated carriers. In addition, the higher concentration of oxygen vacancies in anatase leads to more efficient charge separation, whereas the larger specific surface area leads to more active sites exposure, which are also important factors for its better photocatalytic performance than rutile. However, the narrower band gap enables rutile to respond to photons close to the visible region. Besides, the higher crystallinity results in the better charge carrier mobility within rutile. At present, the integration of the advantages of anatase and rutile to construct a phase heterojunction for photocatalytic CO_2_ reduction has attracted increasing attention of researchers. As a classic anatase-rutile phase heterojunction, the commercial Degussa P25 has been regarded as a benchmark for both photocatalytic oxidation and reduction reactions. As reported by Reñones et al., hierarchical TiO_2_ nanofibres composed of anatase and rutile nanoparticles were synthesized by the calcination of electrospun TiO_2_ fibers under Ar atmosphere (Reñones et al., 2016). The phase composition of the fibers depend on the calcination conditions, in which higher rutile amount (81:19) was obtained under static Ar atmosphere (Fibers B) than the dynamic sample (Fibers A, 93:7). Notably, the faster charge transport along the grain boundaries in fibers is attributed to the improved nanocrystals connection, whereas the fibers with higher anatase content exhibits lower recombination efficiency of *e* ^*-*^/*h* ^*+*^ pairs, thus endowing greater photocatalytic efficiency for reducing CO_2_ to CO (10.19 μmol g_cat_^−1^ h^−1^). In addtion, the overall apparent quantum yields (AQY) of Fibers B (0.036%) is also higher than P25 (0.030%), indicating the enhanced utilization of incident light. However, the large amount of hydrogen evolution (19.94 μmol g_cat_^−1^ h^−1^) during the photocatalytic process needs to be suppressed to further improve the photoreduction efficiency of CO_2_. A hydrothermal method was developed by Xiong et al. for the fabrication of anatase-rutile heterophase TiO_2_ nanoparticles using K_2_TiO(C_2_O_4_)_2_∙2H_2_O as Ti source, which simplifies the synthesis process and makes the reaction conditions more mild (Xiong et al., 2020). Ethylene glycol (EG) was added to the hydrothermal system in order to introduce oxygen vacancy into TiO_2_ (TiO_2-x_), which could trap photogenerated *e* ^*-*^ to restrain the recombination of *e* ^−^/*h* ^+^ pairs, thereby enhancing the photocatalytic performance of the catalysts. Electron paramagnetic resonance (EPR) spectra revealed that the concentration of oxygen vacancy increased with the increasing amount of EG during the formation process of TiO_2-x_. However, the excess oxygen vacancies, especially the bulk oxygen vacancies, will act as the recombination center of carriers, resulting in the lower photocatalytic efficiency of as-obtained anatase-rutile heterojunction. Thus, the concentration and distribution of oxygen vacancies should be reasonably designed and constructed to promote the performance of the semiconductor photocatalysts. As reported by Xiong et al. **(** Xiong et al., 2020 **)**, the TiO_2_-EG10 sample synthesized using 30 ml H_2_O and 10 ml EG as solvent exhibits the optimized photocatalytic activity in reducing CO_2_ to CH_4_ (43.2 μmol g_cat_^−1^ h^−1^), which is 54 times higher than that of P25 (0.8 μmol g_cat_^−1^ h^−1^). Usually, the optimization of oxygen vacancy concentration in photocatalyst is based on the feedback of experimental results rather than theoretical design. Therefore, the critical oxygen vacancy concentration in different photocatalytic systems is various, which is difficult to give a definite value. Considering the poor visible light responsibility of anatase/rutile phase heterojunction, it is necessary to reduce its band gap width to improve the utilization of incoming solar spectrum. In addition, the combination of disordered anatase (A_d_) with more active sites and ordered rutile (R_o_) for fast transport of *e* ^*-*^ and *h* ^*+*^ to suppress charge recombination can further enhance the photoreduction efficiency of CO_2_ by TiO_2_. On this basis, a phase-selective A_d_/R_o_ TiO_2_ was prepared by treating P25 in the sodium alkyl amine solutions at room temperature and ambient atmosphere, in which anatase among P25 was selective reduced to produce more Ti^3+^ defects (Hwang et al., 2019). The existence of multi-internal energy bands of Ti^3+^ defect sites in A_d_ reduces the band gap of A_d_/R_o_ TiO_2_ to 2.62 eV, while the newly conduction band (-0.27 eV) is well match the redox potential of CO_2_/CH_4_ (-0.24 V vs. NHE). As a result, the VLD A_d_/R_o_ TiO_2_ exhibits enhanced reactivity to convert CO_2_ into CH_4_ (3.98 μmol g_cat_^−1^ h^−1^), which is higher than metal (W, Ru, Ag, and Pt)-doped P25. To further improve the CH_4_ generation selectivity, 0.1% mass Pt was loaded on the H_2_O_2_ modified TiO_2_ (M-TiO_2_, containing two phases of anatase and rutile) through photoelectrodeposition (Pt/M-TiO_2_) (Lee J. S. et al., 2016). As electron sinks, photogenerated electrons are enriched by Pt nanoparticles and form high charge density areas near their surface, which is favoring for the photoreduction of CO_2_ to CH_4_ in cooperation with sufficient protons generated by water oxidation. Notably, the yield of CH_4_ on Pt/M-TiO_2_ is about 60 times that of bare M-TiO_2_, whereas no CO formation can be observed, indicating its high selectivity toward photoreduction products of CO_2_. Compared with the common method that fabricates phase heterojunction by calcining TiO_2_ gel, the MOFs (NH_2_-MIL-125) derived method was adopted by Chen et al. (Chen et al., 2020) to fabricate anatase-rutile junction with large surface area and porous structure that favored for CO_2_ adsorption. Besides, the *in situ* phase transformation from anatase (211) plane to rutile (211) plane results in the highly dispersed anatase/rutile interface for efficient interfacial charge separation, inhibiting the recombination of photogenerated *e* ^*-*^/*h* ^*+*^ pairs significantly. Moreover, the N-doped carbon layer (generated by the pyrolysis of organic ligands) coating on the anatase/rutile heterostructure promotes the electric conductivity of the photocatalytic system, and expandes its light absorption range to 700 nm. The synergism of N-doped carbon and paragenetic anatase/rutile heterostructure derives the enhanced photoreduction efficiency of CO_2_ to CO, which is 7.6 folds compared with P25. Although the added value of the product (CO) is limited, this method has guiding significance for the design and synthesis of other MOFs derived heterostructured photocatalytic systems. In addition to rutile, anatase can also form a phase heterojunction with brookite for photocatalytic CO_2_ reduction (Jin et al., 2019). Generally, Sr^2+^ ions were introduced to the TiCl_4_-involed hydrothermal system for the fabrication of SrCO_3_-modified brookite/anatase TiO_2_ heterophase junctions (HPJs). Similar to the anatase-rutile system, the interfacial electron transfer from brookite to anatase promotes the photogenerated charge separation. Moreover, the surface modification of HPJs by SrCO_3_ can improve the adsorption of CO_2_/H_2_O, which can also serve as an efficient cocatalyst for the selective reduction of CO_2_ to CH_4_. Especially, 1.0 w/w% SrCO_3_/HPJs composite shows the selectivity of ca. 7.45 for CH_4_/CO (19.66/2.64 μmol g_cat_^−1^ h^−1^), which is ca. 32.4 folds compared with pristine brookite TiO_2_ (0.79/3.46 μmol g_cat_^−1^ h^−1^). This work can also provide guidance for the development of heterojunctions composed of TiO_2_ HPJs and alkaline earth metal carbonates *via* a facile one pot hydrothermal route.

#### TiO_2_ Based Facet Heterojunction

**FIGURE 12:**
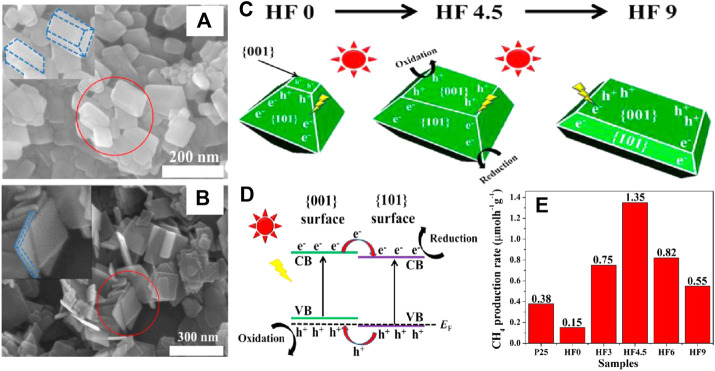
FESEM images of **(A)** HF4.5 and **(B)** HF9; Schematic illustration of **(C)** tuning the ratio of {101} to {001} facets of anatase TiO_2_ by adjusting the amount of HF, and **(D)** charge transfer at the interface of {101}-{001} facet heterojunction of anatase TiO_2_; **(E)** Comparison of the photocatalytic CH_4_-production activity of P25 and the TiO_2_ samples prepared by varying HF amount. Reproduced from Yu et al. (2014) with permission from the American Chemical Society.

The difference of geometrical and electronic structures between different crystal facets of the same semiconductor results in the distinctness of their photocatalytic activity. Facet engineering has been applied to control the exposed crystal facet of semiconductors, in order to increase the exposure of active sites and promote the adsorption and activation of substrates, so as to achieve enhanced photocatalytic performance. In terms of anatase TiO_2_, the {101} has the lowest surface energy (0.44 J m^−2^) among the low-index facets (including {001}, {010} and {101}) and dominant for CO_2_ adsorption basing on first-principles calculations (Yu et al., 2014). The photogenerated electrons transferred from the {101} of TiO_2_ to CO_2_ facilitates its activation and reduction. Moreover, the enriched photogenerated holes on the {001} of TiO_2_ can accelerate the oxidation reactions. Photocatalytic CO_2_ reduction over anatase TiO_2_ with coexposed {001} and {101} facets was reported by Yu et al. for the first time with the propose of “facet heterojunction” concept (Yu et al., 2014). The facet ratio of {001} and {101} can be tuned by adjusting the amount of HF during the fabrication process, as see in Figures 12A,B, while the correspondingly schematic illustration is displayed in Figure 12C. In particular, the formation of facet heterojunction between {001} and {101} contributes to the transfer and separation of photogenerated carriers, which is beneficial to the enrichment of *e* ^−^ and *h* ^+^ on {101} and {001}, respectively (shown in Figure 12D). As a result, photocatalytic CO_2_ reduction occurs selectively on the {101} facet, while the optimized photocatalytic performance is realized when the facet ratio of {101} to {001} is 45–55, in which CO_2_ is reduced to CH_4_ with a generation rate of 1.35 μmol g_cat_^−1^ h^−1^ (Figure 12E). On this basis, oxygen vacancies were introduced to the {101} and {001} facets coexposed anatase TiO_2_ for the further enhancement of photocatalytic performance, where TiO_2_ fabricated *via* hydrothermal route in the presence of HF was reduced by NaBH_4_ to generate surface oxygen defects (Liu L. et al., 2016). In addition to the high charge separation efficiency at the interface of {001} and {101} facets, the visible light responsibility attributed to the newly generated Ti^3+^ energy state as well as the improved CO_2_ adsorption and activation derived from the synergism of Ti^3+^ and oxygen vacancies resulted in the enhanced photoreduction efficiency of CO_2_. An explicit atomistic model of the interface was applied for further investigation of charge transfer between coexposed {101} and {001} facets of anatase TiO_2_ (Di Liberto et al., 2019). The first principles calculations revealed that the localization of *h* ^+^ on oxygen ion of the {001} side and the migration of *e* ^−^ to Ti ion of the {101} side promoted the charge separation and suppressed their recombination, hence responsible for the enhanced photocatalytic activity of the facet junction. In another work, HF was also used by Cao et al. (Cao et al., 2016) for tuning the ratio of coexposed {101} and {001} facets of anatase TiO_2_ in a hydrothermal system where nanotube titanic acid (NTA) was used as precursor to facilitate the mass transfer of HF, thereby simplifying the generation of {001} facet. Notably, the anatase TiO_2_ nanocrystals with coexposed 51% {001} and 49% {101} facets exhibit the highest photocatalytic activity for reducing CO_2_ to CH_4_ (1.58 μmol g_cat_^−1^ h^−1^) among pure TiO_2_ series, which can be further improved to 4.0 μmol g_cat_^−1^ h^−1^ by decorating 1wt% Pt^0^ nanoparticles on their surfaces. The effect of Pt loading for the enhanced photocatalytic performance of anatase TiO_2_ facet heterojunction ({101}/{001}) was further investigated by Xiong et al. (Xiong et al., 2017a). Pt precursors (H_2_PtCl_6_ or Pt (NH_3_)_4_Cl_2_) and deposition methods (photodeposition or chemical reduction) had significant influence on size, distribution, and valence states of Pt nanoparticles, hence led to the difference of photocatatic performance. In particular, the well dispersed Pt nanoparticles fabricated by chemical reducing H_2_PtCl_6_ (HC) possessed suitable particle size and high Pt^0^/Pt^II^ ratio (1.15), which led to efficient separation of photogenerated carriers and high efficiency for photoreduction CO_2_. Coupling with graphene is also an efficient strategy to enhance the activity of anatase TiO_2_ facet heterojunction ({101}/{001}) for the photoreduction of CO_2_ (Xiong et al., 2016). Charge transfer between {101} and {001} facets along with the migration of electrons from TiO_2_ to graphene greatly improved the separation efficiency of photogenerated carriers, thus increasing the yield of CO by photoreducing CO_2_. Photocatalytic CO_2_ reduction performance of the typical TiO_2_-based phase or facet heterojunctions above are listed in Table 3.

## Conclusions and Prospects

This review summarizes the recent advances in the rational design, fabrication and photocatalytic performance of TiO_2_-based heterojunctions for converting CO_2_ into solar fuels with water oxidation. Generally, photocatalytic CO_2_ reduction that mimic the nature photosynthesis of green plants exhibits great potential for the reduction of CO_2_ level in the atmosphere and storage of solar energy in hydrocarbon fuels, so as to alleviate the impact of energy crisis and climate change on the development of human society. However, some obstacles, such as low solar energy conversion efficiency, slow generation rate and poor selectivity toward reduction products of CO_2_, and common photocorrosion phenomenon facing the current photocatalytic systems, restricts the practical application of this very promising technology. In recent years, tremendous efforts have been devoted to fabricate TiO_2_-based heterojunctions, in order to realize enhanced photocatalytic performance for CO_2_ conversion, thus giving new life to this traditional and systematically studied photocatalyst. Although the composition, morphology, architecture and photocatalytic mechanism of TiO_2_-based heterojunctions are various, they have much in common that favors for photoreduction of CO_2_ as follows: 1) the efficient electron transfer at the heterointerface that promotes spatial separation of photogenerated *e* ^−^/*h* ^+^ pairs and prolongs their lifetime to participate in the photoinduced redox reactions; 2) the expanded light adsorption range and enhanced visible light responsibility, making it possible for solar-driven photocatalytic CO_2_ reduction; 3) the enlarged CO_2_ adsorption capacity due to the high specific surface area with highly exposed active sites that combine CO_2_ by chemical action, which could also activate the adsorbed CO_2_ molecules and facilitate hydrocarbon generation; 4) the increase in selectivity toward specific photoreduction products of CO_2_ is attributed to the contribution of the cocatalysts.

Although considerable progress has been made on TiO_2_-based heterojunction for photocatalytic CO_2_ reduction, it is still far from practical application. On the one hand, the formation of multi-carbon products has always been a bottleneck in this field. The study of CO_2_ photoreduction intermediates combined with *in-situ* analysis technology and theoretical calculations needs to be more in-depth in order to clarify the formation mechanisms of different hydrocarbons and guide for the rational design of photocatalysts for the generation of multi-carbon product with high selectivity. On the other hand, pre-defined design of the components and their spatial arrangement in the heterojunction for the optimized phtotcatalytic performance is still a great challenge. It is worth nothing that the difference in synthesis conditions limits the flexibility of component selection, which also complicates the synthesis procedure and reduces the yield of expected heterojunction photocatalyst. At the same time, the randomness arrangement of different components in many cases and the variability of the catalyst morphology and structure affect the photocatalytic performance significantly, which also makes it difficult to clarify the contribution of each component and the synergism mechanism. How to overcome the above limitations to select components of the heterojunction based on photocatalytic performance only, and achieve precise control at the structural unit level, thus realizing efficient synergy of each component for photocatalytic CO_2_ reduction is one of the main directions of future development in this field. To our knowledge, recent advances in DNA origami superlattice structure (Tian et al., 2016; Tian et al., 2020) may provide a possible solution and guide the design and construction of well-ordered heterojunction photocatalysts in the future. Specifically, the precisely control of the topological structure, arrangement sequence, and assembly quantity of each building block (polyhedral DNA frame) during the self-assembly process makes it possible for heterojunction photocatalysts with controllable structure and adjustable performance, which is expected to become the future research hotspot in the field of photocatalytic CO_2_ reduction. In addition, efficient solar harvesting systems are in great demand to replace artificial light sources with high energy consumption, since efficient photoreduction of CO_2_ is based on high light intensity. On the one hand, upconversion quantum dots can be introduced into the heterostructured photocatalyst, in which some of the long-waved visible light in the incident light can be converted into short-waved partial that can excite the photocatalyst to generate *e* ^−^ and *h* ^*+*^ pairs. On the other hand, focusing lens system can be added to the photoreactor to enhance the concentration of sunlight, then the photocatalyst can operate under higher light intensity and exhibit optimized activity. Although the efficiency of photocatalytic CO_2_ reduction is far less than that of electrocatalysis, relying entirely on solar energy will become its irreplaceable advantage. Furthermore, the enrichment of CO_2_ in air will become an important consideration in the design of heterojunction photocatalysts, which meet the needs of practical applications. Fortunately, the research on CO_2_ storage and controlled release provides the possible solution while the visible light-triggered capture and release of CO_2_ from stable MOFs become the most promising candidate (Park et al., 2011; Li et al., 2016; Lyndon et al., 2015). Obviously, combination of photocatalyst and the above MOFs can realize the efficient recycling of CO_2_ and improve the efficiency of its conversion into solar fuel, which is expected to become a research hot spot in the future.

In summary, the heterojunction photocatalysts with well-organized structure, optimized solar energy conversion efficiency, ideal turnover frequency of CO_2_, and high reduction product selectivity are still the direction of efforts in the future. We hope that this review can inspire new ideas to guide the design and synthesis of high-performance photocatalysts for photoreduction of CO_2_ into solar fuels, thus accelerating the industrialization process of this very promising technology and providing practical help to alleviate energy and environmental crises.
